# Differential gene expression in irradiated potato tubers contributed to sprout inhibition and quality retention during a commercial scale storage

**DOI:** 10.1038/s41598-024-58949-0

**Published:** 2024-06-12

**Authors:** Sanjeev Kumar, Nilantana Bandyopadhyay, Sudhanshu Saxena, Sachin N. Hajare, Varsha More, Jyoti Tripathi, Yogesh Dahia, Satyendra Gautam

**Affiliations:** 1 Food Technology Division, Bhabha Atomic Research Centre, Mumbai, 400 085 India; 2 Natural Storage Solutions Private Limited, Gandhinagar, 382 729 India; 3 Homi Bhabha National Institute, Mumbai, 400 094 India

**Keywords:** Sprout inhibition, Gamma irradiation, Gene modulation, Physiological changes, Economical feasibility, Biochemistry, Physiology

## Abstract

Current study is the first ever storage cum market trial of radiation processed (28 tons) of potato conducted in India at a commercial scale. The objective was to affirm the efficacy of very low dose of gamma radiation processing of potato for extended storage with retained quality and to understand the plausible mechanism at the gene modulation level for suppression of potato sprouting. Genes pertaining to abscisic acid (ABA) biosynthesis were upregulated whereas its catabolism was downregulated in irradiated potatoes. Additionally, genes related to auxin buildup were downregulated in irradiated potatoes. The change in the endogenous phytohormone contents in irradiated potato with respect to the control were found to be correlated well with the differential expression level of certain related genes. Irradiated potatoes showed retention of processing attributes including cooking and chip-making qualities, which could be attributed to the elevated expression of invertase inhibitor in these tubers. Further, quality retention in radiation treated potatoes may also be related to inhibition in the physiological changes due to sprout inhibition. Ecological and economical analysis of national and global data showed that successful adoption of radiation processing may gradually replace sprout suppressants like isopropyl *N*-(3-chlorophenyl) carbamate (CIPC), known to leave residue in the commodity, stabilize the wholesale annual market price, and provide a boost to the industries involved in product manufacturing.

## Introduction

India is the second largest producer (~ 50 million metric tons; MMT) of potato (*Solanum tuberosum* L.) and contributes to 14% of the world’s annual production^1^. Better post-harvest management of potato tubers is required to reduce its losses, maintaining sustained supply and cost stability in the country. The major quality deterioration in potato tuber initiates with sprouting which is followed by moisture and textural losses, rotting and biochemical changes including build-up of reducing sugar^2^. All these changes make the tubers unsuitable for processing by food industries in the varied products including chips and fries; and, also taste of table purpose products are not well appreciated by the consumers^3^.

In potato tubers, there is absence of visible bud growth and cessation of meristematic activity during dormancy stage^4^. The duration of dormancy of tuber largely depends on the genetic makeup. However, it is also affected by preharvest and postharvest management practices and storage conditions^5,6^. Once dormancy breaks, metabolic changes do happen resulting in sprouting. These changes are supposed to be under precise genetic regulations^7–9^. Changes in the level of endogenous phytohormones have been found to play a crucial role in the regulation of dormancy and bud break^6,10^.

Physical sprout suppression has been reported with ultraviolet (UV-C) radiation, high pressure treatment (100 MPa) and high magnetic field (330–350 mT)^11–13^. However, all these methods have severe limitations in commercial deployment with bulk commodity such as potato. Unlike ionizing radiation like gamma, microwave (2.45 GHz) radiation has been reported to induce sprouting in potato tubers^14^. Ionizing radiation such as gamma ray from radioisotopes (Co^60^, Cs^137^) as well as X-ray (< 7.5 MeV) and E-beam (< 10 MeV) from machine sources have been approved by international statutory organizations including IAEA, FAO and WHO for food processing applications at comparatively higher doses, besides induced mutagenesis at comparatively lower doses in agricultural crops to enhance genetic variability for betterment of yield and quality^15^. It has been reported to extend the shelf life of tubers by effectively inhibiting sprouting while retaining processing qualities. Additional advantage of radiation processing of potatoes is that it is recommended to be stored at higher temperature (14 ± 1 °C) compared to that practiced in routine cold storages^16,17^.

It is worth mentioning that storage at low temperature (2–4 °C) prevents sprouting, however it causes cold induced sweetening (CIS)^17^. This makes such potato tubers unsuitable for processing while negatively affecting the consumers’ likeness for table consumption. Such sweetening of stored potatoes leads to non-enzymatic Maillard reaction resulting in appearance of brown discolouration of the cooked or processed products^17^. Maillard reaction is also involved in production of Acrylamide, a toxic compound and possible carcinogen is formed in starchy foods like potato tubers when heated at high temperature. Reducing sugar and amino acid asparagine play a major role in the formation of Acrylamide. Thus, higher reducing sugar content increases the probability of acrylamide formation. Therefore, radiation treatment and storage at comparatively higher temperature of 14 °C also reduces the probability of acrylamide formation in deep fried potato products^18^. Additionally, non-enzymatic ‘After Cooking Darkening’ (ACD) in tubers occurs due to oxidation of colourless ferrous-phenolic complex formed during cooking process^19^. Another methodology is to store potatoes for long period (5–6 months) is based upon treatment with chemical sprout suppressant, i.e., isopropyl *N*-(3-chlorophenyl) carbamate (CIPC or Chlorpropham) and subsequent storage at relatively higher temperatures (8–12 °C). However, use of CIPC is being phased out by many countries due to possible health safety and environmental issues because CIPC and its toxic residue such as 3-chloroaniline (3-CA) and other metabolites have been reported (~ 45 mg/kg) in tubers, potato crisps, French fries and extruded potato peels. Besides, a number of hazardous and harmful metabolites like isopropyl *N*-4 hydroxy-3-chlorophenyl carbamate; isopropyl-*N*-5-chloro-2-hydroxyphenyl carbamate and 3, 3′-dichloro azobenzene have been reported to be synthesized after ingestion of CIPC^20–23^.

Despite these limitations, a question still arises regarding addressing the issue of quality retention of potato tubers during prolonged storage.

In this context, radiation technology could provide a global solution to spoilage issue in potatoes. Japan is using radiation processing of potatoes since 1973 and ~ 5767 tons were processed during 2015 but this has not well spread in other countries^24^. Based upon the research data, Government of India (GOI) approved radiation processing of potatoes in 1994 for sprout inhibition. Subsequently in 2016, a class-based approval was granted by GOI and Gazette notified through FSSAI (Food Safety and Standards Authority of India) where irradiation of potato tubers was kept in class I which include bulbs, stem and root tubers, and rhizomes^25^. The absorbed dose for sprout inhibition was approved in the range of 20–200 Gy^25^. In spite of this, radiation processing of potato tubers in worldwide including India has not gained expected momentum. One of the possible reasons could be lack of suitable business model for this low-cost commodity although its consumption is widespread across the globe. Furthermore, mechanism of sprout inhibition and quality retention of potato tubers upon treatment with ionizing radiation is still obscure.

The purpose of the current study was to demonstrate the efficacy of gamma radiation treatment in suppression of sprouting in potato tubers and revealing the underlying molecular mechanism primarily by evaluating the modulation of expression of genes related to growth, cell division, central dogma as well as carbohydrate metabolism. Though, there are plenty of scientific reports on sprouting-inhibitory effect of ionizing radiation; a combined model based on molecular mechanism is essential for better understanding of the technological insight. This would be very helpful in dissemination of the radiation technology particularly for the treatment of popular chip making potato varieties for commercial purposes. Secondly, the study was scaled up to a commercial level (28 tons) for assessing the feasibility of industrial application in agriculture dominated country like India and its economic sustainability at the prevailing infrastructure. The outcome of the study establishes the feasibility of potato tuber irradiation at industrial scale and specifically designates the ideal storage temperature and humidity for three most important processing varieties of potato in India. Moreover, the results draw a realistic conclusion on the quantum of post-harvest losses in potatoes even being stored in the cold storages and quantitates the advantage of radiation treatment in terms of weight loss, quality retention, economic viability, and market stabilization at the industrial scale.

## Methods

### Selection of varieties for large-scale commercial trial

**Figure 1:**
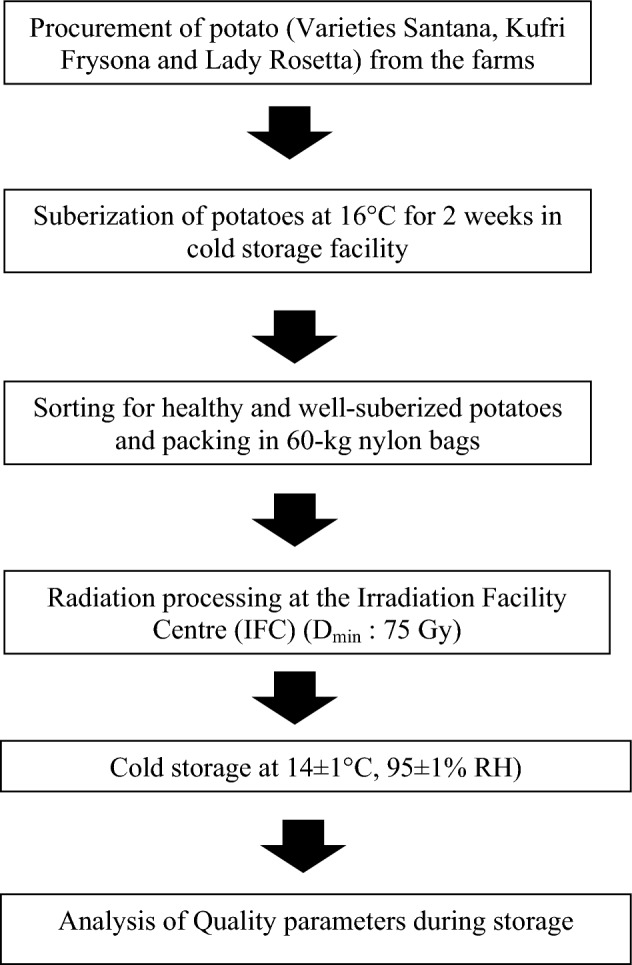
Schematic representation summarizing the potato irradiation, preservation and quality assessment.

Three prominent commercial varieties of potato were selected in the current study. These varieties included ‘Santana’, ‘Kufri Frysona’ and ‘Lady Rosetta’. Santana tubers are high yielding, large, oval in shape and popular for frying due to its attractive cream-coloured interior. It was developed in Netherlands in 1994 from Spunta. Kufri Frysona, an oblong high yielding tuber was developed in 1998 by Central Potato Research Institute at ‘Kufri’, India. It is a hybrid obtained from the cross between MP/92–30 × MP/90–94 and has ideal oblong shape, colour and texture for making French fries^26^. Lady Rosetta, a uniform round red skinned, long-dormancy variety was developed in Netherlands in 1988 from cross of CARDINAL x VTN 62-33-3, and it has been popular in the Europe and Middle East for its excellent chip making properties^27^. A schematic representation summarizing irradiation, preservation and quality assessment of potato tubers has been displayed in Fig. 1.

### Procurement and optimization of process parameters for preservation of potato tubers

All plant experiments were carried out in accordance with relevant national and international guidelines and regulations. 28 tons of potatoes (Santana: 8 Tons, Kufri Frysona: 12 Tons and Lady Rosetta: 8 Tons) were procured in March 2021 from potato farms at Gujarat, India. The study was performed in joint collaboration with a private firm, Natural Storage Solutions Private Ltd (NSSPL), Gujarat, India through a memorandum of understanding (MoU). For proper suberization, potatoes were placed at 16 °C for 2 weeks in cold storage facility at Maharashtra State Agricultural Marketing Board (MSAMB), Navi Mumbai, Maharashtra, India. Healthy and well-suberized potatoes were packed in 60-kg nylon bags, from these, half of each variety (Santana: 4 Tons, Kufri Frysona: 6 Tons and Lady Rosetta: 4 Tons) was irradiated (I) at Irradiation Facility Centre (IFC), MSAMB, while the rest served as non-irradiated control (C). Potatoes (exposed to various radiation doses and control samples) were stored at various temperatures (26 ± 2, 14 ± 1 and 3 ± 1 °C) and relative humidity (RH; 85 and 95%) and periodically assessed for sprouting, rotting and desiccation/moisture loss. Thus, process parameters were optimized for determining the most suitable storage conditions for better preservation while retaining cooking/ chip-making qualities.

### Radiation processing and storage

Average radiation dose was measured as 106 Gy where D_*min*_ was 72 Gy and D_*max*_138 Gy using a cobalt-60 source as per the approved guidelines^28^. Dosimetry of the irradiator was conducted using a standard Fricke dosimeter. The dosimeters were placed at all the three (front, middle, and rear vertical) planes of the tote box. Absorbed dose was derived by measuring the ferric ions concentration spectrophotometrically at 304 nm. Irradiated potatoes were transported through a reefer container to a cold storage facility (storage chamber conditions: 14 ± 1 °C, 95 ± 1% RH) located at Mehsana, Gujarat. During storage potatoes were monitored for sprouting and rotting losses as well as quality retention.

### Physical, biochemical and nutritional quality attributes

Ten bags (30 kg each) of these varieties were weighed periodically during storage to determine the weight loss (%). Textural quality was analyzed by a Texture Analyzer (TA.HD plus, Stable Micro Systems, Godalming, Surrey, UK) with a P/2N needle probe and expressed as the force (*g*)^17^. It measures the hardness of the sample based on resistance offered to the penetrating needle probe^17^. Colour analysis inside potato tuber was carried out using a colorimeter (Konica Minolta Sensing Inc., CM-3600d, Osaka, Japan; JAYPAK4808 software, Quality Control System, Version 1.2). Multiple reflectance values in the range of 360–780 nm were recorded at an interval of 10 nm from each tuber and at least three tubers were used for each variety. Values were transformed to give rectangular colour coordinates (L*: Lightness, a*: + red/ −green, b*: + yellow/ − blue) as well as chroma and hue angle. Colour difference between the samples were calculated as $\Delta {\text{E}} = \sqrt {} \left[ {\left( {{\text{L1}}\, - \,{\text{L2}}} \right)^{{2}} \, + \,\left( {{\text{a1}}\, - \,{\text{a2}}} \right)^{{2}} \, + \,\left( {{\text{b1}}\, - \,{\text{b2}}} \right)^{{2}} } \right]$. Reducing sugar content was estimated by the dinitrosalicylic acid (DNSA) method^17^.

Nutritional parameters were analyzed in terms of energy value, carbohydrate content, protein and fat^29^. Protein content was determined as per the Bureau of Indian Standards by Kjeldahl analysis^30^. Total fat content was determined after extracting in petroleum ether in a Soxhlet extraction apparatus as per the Bureau of Indian Standards^31^. Total vitamin C (ascorbate) content was determined by titration using 2,6 dichlorophenol indophenol (DCPIP) dye^32^. The total vitamin B6 was quantified by ion-pair liquid chromatography (LC). Here, vitamin B6 was dephosphorylated (enzymatic hydrolysis), reacted with glyoxylic acid (Fe^+2^ catalysts) where pyridoxamine gets converted to pyridoxal which was subsequently reduced to pyridoxine by the action of sodium borohydride in the alkaline medium^33^. Energy content was estimated by standard calculation based on the protein, sugar, fat and carbohydrate contents of the sample. Carbohydrate content was determined by deducting the percentage values (g/100 g) of moisture, ash, protein, and fat from 100. Potassium content was determined by flame emission spectrometric method^34^.

### Transcriptome analysis of potato tubers using RNA sequencing

To understand the mechanism of gamma radiation induced sprout inhibition at cellular level in these potato varieties, the gene expression profile of irradiated and non-irradiated control potatoes was carried out using RNA-seq analysis after 90 days of storage. This time point was selected as complete sprouting was observed in control potatoes of varieties under study. Impact of differential expression due to radiation processing of potato tuber on quality retention was also analyzed. RNA-seq analysis involved RNA isolation, library preparation, sequencing and bioinformatics analysis.

### RNA isolation and library preparation

For RNA-seq analysis, 10 kg of samples were randomly selected as the ‘initial material’. The variation in individual tuber was overcome by pooling in tubers followed by homogenization and RNA extraction in duplicate for all the three varieties (control and radiation treated stored) sample. Similar approach was adopted in previous study too^35^. Briefly, RNA was extracted from 100 g of potato tissue homogenate (frozen in −80 °C) using Trizol reagent as per the manufacturer’s protocol (Sigma, USA). Poly(A) RNA was captured with magnetic oligo-dT beads (KAPA mRNA HyperPrep Kit, Roche, USA). First strand cDNA was synthesized using random primers. 2nd strand cDNA synthesis converted cDNA:RNA hybrid to double-stranded cDNA (ds cDNA), while marking the 2nd strand with dUTP. dAMP was then added to the 3′-end of ds cDNA fragments. 3′-dTMP adapters were ligated to 3′-dAMP library fragments. Adapter-ligated library DNA was amplified by polymerase chain reaction (PCR). Library fragment size distribution was confirmed by electrophoresis. Library concentration was determined by qPCR (KAPA Library Quantification Kit, Roche, USA).

### RNA sequencing and bioinformatics analysis

Further, Illumina NovaSeq 6000 was utilized for Paired End Sequencing (read length 2 × 150). Quality control of sequence reads was performed using Fastp software included within the Illumina sequencer, where reads having < 1% error rate were considered (Q 20) and duplicate reads, poor quality sequence were automatically discarded. Read quantification was done by Feature count Rsubread Package (version 2.0.3). Alignment of filtered reads was done using Hisat 2 (version-2.1) genome assembler onto the *Solanum tuberosum* reference genome GCA_000226075.1^36^.

Differential expression analysis was performed using statistical methodology included in EdgeR (Version: R 4.2 version) package where transcripts having expression equal to or more than 1 reads per million kilobase were considered for assembly. Functional annotation clustering was done using DAVID bioinformatics tool (Database for Annotation, Visualization, and Integrated Discovery, LHRI). GO enrichment analysis was based on UniProt database and GO terms were placed under molecular function, biological process and cellular compartmentalization categories using g: Profiler: a web server for functional enrichment analysis and conversions of gene lists. Afterwards relevant GO terms for upregulated and downregulated (differentially expressed) genes (DEGs) of radiation treated tubers from all three varieties were displayed using bubble plot. DEGs having similar change in expression profile (upregulated, downregulated or neutral) in all the three varieties upon radiation treatment were selected and their functional analysis was carried out to produce a proposed model depicting effect of radiation treatment on shelf-life extension of potato tubers. Expression profile of genes known to be involved in carbohydrate metabolism was further analyzed using web enabled expression-based Heat mapper tool.

### Phytohormone quantification

Phytohormones [Auxin (Indole-3-acetic acid; IAA), Gibberellic acid (GA3) and Abscisic acid (ABA)] were extracted from potatoes using standard methods adopted from previous literature as discussed below. For estimation of IAA, lyophilized (1 g) potato tuber powder was extracted thrice with acidified methanol (pH 2.5; 5.5 mL). The extract was filtered and the total volume was made upto 20 mL. The solvent was evaporated to dryness and resuspended in methanol (1 mL). Aliquots of this extraction was subjected to thin-layer chromatography (TLC; Chloroform: ethylacetate: formic acid = 77:22:1, silica gel 60-F254, Merck, US) along with suitably diluted standard IAA (Sigma-Aldrich, St. Louis, MO, USA)^38^ and quantified using densitometry (CAMAG HP-TLC system equipped with in WinCATs software and Image J). Similarly, for free GA3, the lyophilized powder was extracted using aqueous methanol (80%), followed by extraction with ethylacetate and methanol (50%,v/v). TLC was carried out using a solvent system of isopropanol: ammonia: water = 10:1:1^39^. ABA was extracted in 80% acetone, dried and dissolved in methanol before performing TLC in solvent system comprising of toluene: ethylacetate: formic acid: methanol (3:3:0.8:0.2)^40^.

### Cooking quality assessment

Potatoes were cooked under pressure at around 121°C followed by Browning index (BI) determination for assessing the darkening impact of cooking. Browning factors were extracted from cooked and mashed potatoes (1.5 g) using 15 mL ethanol (67%) for 1 h, filtered (Whatman 1 paper) and quantified using a spectrophotometer at 420 nm^41^.

### Sensory evaluation of processed product

Processing quality of potatoes was assessed by deep frying of tuber slices in oil at nearly 180 °C^2^. The slices of potato tubers were prepared using hand operated slicer or chip cutter. Prepared chips were evaluated by the panel of taste panellists (18 numbers) in the age group of 25–50 years for acceptability^17^. The quality parameters (appearance, colour, texture, odour, taste and overall acceptability) were evaluated on a 9-point hedonic scale (9 = “like extremely”, 8 = “like strongly”, 7 = “like very well”, 6 = “like fairly well”, 5 = “like moderately”, 4 = “like slightly”, 3 = “dislike slightly”, 2 = “dislike moderately” and 1 = “dislike extremely”)^42^.

### Statistical analysis

Statistical analysis of samples was carried out by OriginPro software, Version 8.5.0 SR1 (OriginLab Corporation, Northamton, MA USA). Each experiment was repeated in three different sets and the parameters like mean and standard deviation were calculated for all the observations. Mean along with standard deviation were represented for all the observations. Variation observed due to treatment and storage was analyzed using One-way analysis of variance (ANOVA). To test the normality of the data, Shapiro–Wilk test was employed with p ≤ 0.05 level of significance, while the Homogeneity of Variance was analyzed by applying Brown-Forsythe test with p ≤ 0.05 level of significance. The data sets followed normal distribution and had a homogenous variance based on the results of these tests. For means separation test, Fisher’s least significant difference (LSD) was used and all the analyses were carried out at p ≤ 0.05 level of significance. Correlation value was determined between mean differential gene expression levels of relevant genes and change in phytohormone or sugar/vitamin B6 content (radiation treated vs. control) of all the three varieties using ‘CORREL’ function of Microsoft Excel.

## Results and discussion

Potato tuber has been used as staple food in countries like Peru, Bolivia, Argentina, Chile, and dietary ingredient in UK, USA and Indian subcontinent^43^. It is shown that potato cultivation produces less green house gas and requires less irrigation compared to major crops such as wheat and maize; leading to the promotion of the potatoes as staple food in countries like China^44^. There is an issue of short dormancy period in potato which has been attempted to resolve earlier through integration of different post-harvest practices^45^. One such approach was storage at low temperatures where hormone biosynthesis required to break dormancy remain suppressed^46^. However, this was not found to resolve the issue completely as withdrawing led to profuse and quick sprouting across the potato varieties^47^. Besides, certain cold induced enzymes like amylases get activated leading to increased sweetening^48^. This not only adversely affected the sensory quality but also its industrial application as processed products. Many such products are prepared by deep frying at high temperature, where, increase in reducing sugar often leads to undesirable browning due to Maillard reaction^41^. Thus, the issue still remains unresolved to develop a most suitable, reliable and safer storage method.

Therefore, radiation processing was investigated to establish its efficacy in addressing the above said challenges at commercial scale. For comparatively low-cost agri-commodity such as potatoes, a large scale trial needs to be conducted for evaluating its commercial viability. In parallel, quality attributes including in-depth understanding needs to be established for scientific reliability and wider adoption by industry as well as consumers. Three important potato varieties, namely, Santana (cream to pale yellow colour), Kufri Frysona (white colour, high disease resistance) and Lady Rosetta (slight reddish colour) considered suitable for processing by industries were selected. Freshly harvested tubers were cured for 2 weeks prior to radiation treatment.

### Optimization of process parameters for better preservation of potato tubers

A preliminary study was conducted to optimize the storage condition. The study revealed that storing potatoes (radiation treated as well as control one) at ambient temperature (26 ± 2 °C) resulted in desiccation resulting in drastic quality deterioration. Besides, occasional rotting also started upon prolonged storage after 4 months. On the other hand, irradiated potatoes stored at 14 ± 1 °C and RH of 95 ± 1% were found to retain all the qualities with insignificant change in moisture content. However, profuse sprouting and weight loss was observed in control samples. Further, build-up of reducing sugar was not observed in irradiated samples, but the same was higher in control samples due to sprouting. Storing at further lower temperature of 2–3 °C have been reported to cause cold induced sweetening due to activation of vacuolar invertase, UDP-glucose pyrophosphorylase and amylase^49–51^. Therefore, detailed study was performed at large scale with radiation processed potato tubers stored at 14 ± 1 °C at RH of 95 ± 1%. Lower RH did not serve the purpose due to enhanced moisture loss and shrinkage. A minimal absorbed gamma radiation dose of 73 Gy served the desired purpose of complete inhibition of sprouting while retaining the quality attributes. Depending upon dose uniformity ration (DUR) of the radiation facility the dose varied between 73 and 138 Gy. This absorbed dose range also suffice the recommendation as per the ‘Food Irradiation Rule’ endorsed by FSSAI (Food Safety and Standards Authority of India), Government of India vide Gazette notification^52^.

### Effective inhibition of sprouting upon radiation treatment

**Figure 2:**
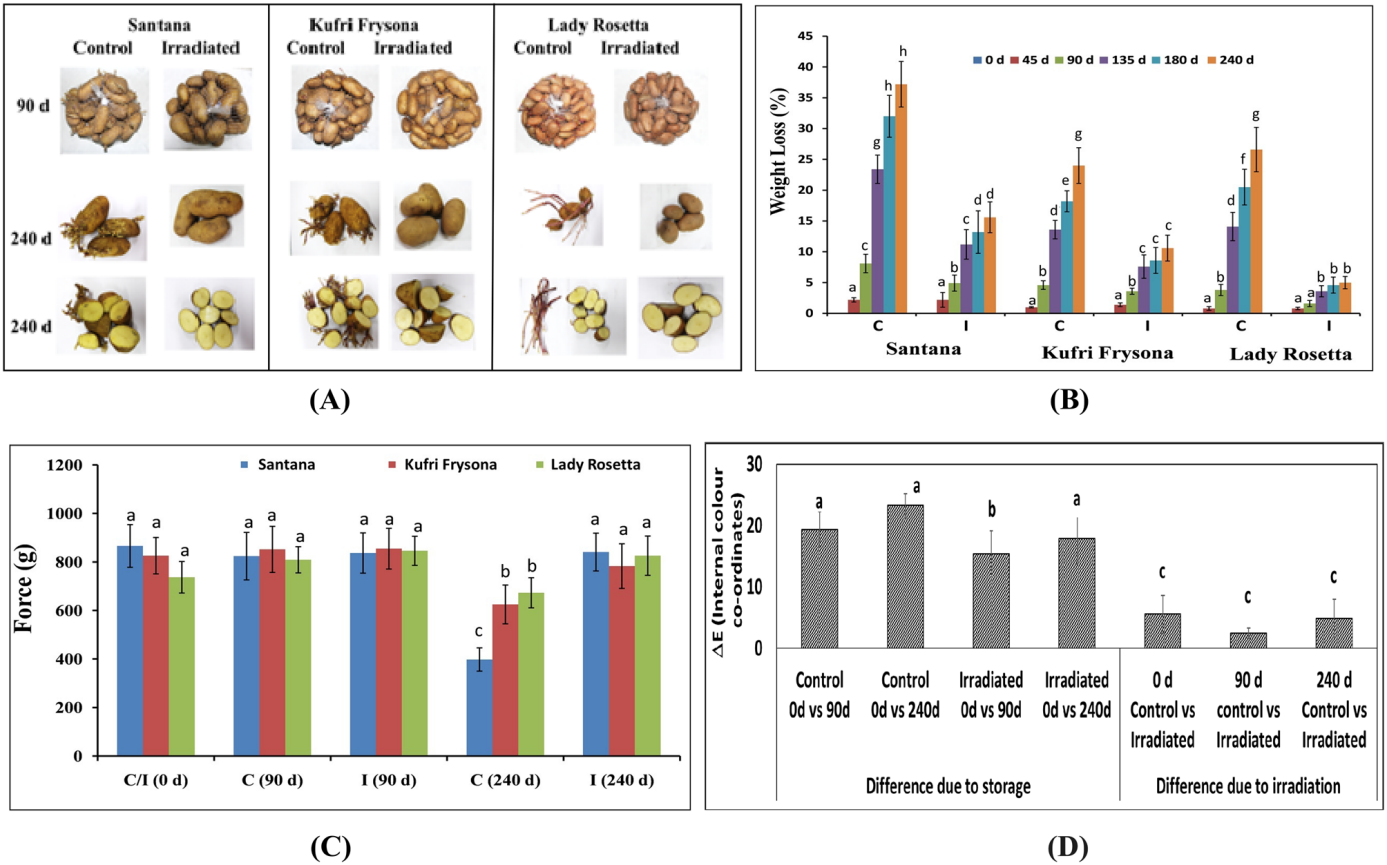
Sprouting of control potato and visual internal quality during storage (**A**). Evaluation of weight loss (**B**), textural quality (**C**) and overall total internal colour difference (ΔE) (**D**) of control and radiation irradiated potato tubers during storage. Different letters on the top of the bars indicate statistically significant difference among means (p ≤ 0.05).

In control potatoes, 50–65% samples were found to be sprouted after 45 days of storage (Table S1). However, complete sprouting (100%) was observed in all the three varieties after 90 days of storage (Table S1). The sprout length for Santana, Kufri Frysona and Lady Rosetta after 90 days was 4.9 ± 1.4, 3.7 ± 0.8 and 5.9 ± 3.4 cm, respectively (Table S1). Once sprouted, potatoes were found unsuitable for industrial processing as well as even not acceptable for table consumption. However, sprouting was completely inhibited in radiation processed potatoes till 240 days of storage for all the three varieties. Due to profuse sprouting, complete quality deterioration leading to spoilage was observed in control tubers (Fig. 2A). Only 3–4% rotting was observed in the irradiated samples even after 240 days of storage.

### Radiation treatment aided physical quality retention in potatoes

Weight loss is an important commercial parameter. After 45 days of storage, weight loss in control and irradiated samples ranged from 3.8 to 8.1 and 1.6 to 4.9%, respectively (Fig. 2B). However, it became pronounced during further storage. At the end of 240 days, tuber weight was found to decrease further and the weight loss ranged between 24 and 37% in control samples but only between 5 and 15% in irradiated tubers depending upon variety. Primary advantage of radiation treatment of tubers was clearly indicated in the periodic weight loss data of these varieties (Fig. 2B)^5^. These findings further indicated that in irradiated Lady Rosetta variety weight loss was the minimum. It is speculated that during sprouting higher rate of metabolism of stored starch and protein including transpiration losses led to more weight loss in control potatoes^53^.

As the sprouts grew, progressive increase in metabolism caused substantial weight loss of tuber ultimately resulting in further shrinkage and textural losses. Texture (hardness) of fresh (0 day) non-irradiated control or irradiated samples of these varieties was in the range of 737–866 g force. During storage of 240 days, texture of control samples became softer and texture value declined significantly to 398–673 g force, which was found to be well retained (783–841 g force) in the radiation treated one (Fig. 2C).

Internal colour assessment was carried out to check for colour difference (ΔE) based on the parameters L, a, and b as discussed above. This was performed for (i) control and irradiated samples at specific storage time (0, 90 and 240 days), as well as (ii) across different storage time points (between 0 and 90 days, between 0 and 240 days) separately, for control and irradiated tubers. The colour co-ordinate values, as well as, ΔE values of control and radiation treated tubers belonging to all three varieties at different time points has been elucidated in Table S2A,B. The data indicated that irrespective of variety the effect of storage is more prominent than the effect of radiation treatment. Therefore, the overall colour (∆E values averaged from all three varieties) regarding storage and irradiation has been depicted in Fig. 2D. From the results (Fig. 2D), it is evident that ΔE between non-irradiated control and irradiated samples at any specific time point is quite less. The average ΔE _(C vs. I)_ for all varieties at 0, 90 and 240 days’ were 5.6, 2.5 and 4.9 respectively. Further, when ΔE was calculated again between two different storage points, the values were considerably higher. ΔE of control samples between 0 and 90 days (ΔE _0d vs. 90d_) was 19.4 and between 0 and 240 days (ΔE _0d vs. 240d_) was 23.2. In radiation treated tubers the values for ΔE between 0 and 90 days _(0d vs. 90d)_ was 15.4 and that between 0 and 240 days (ΔE _0d vs. 240d_) was 17.9, respectively. Therefore, it can be concluded that the overall colour difference of potato tuber is mostly influenced by storage duration. Moreover, treatment with radiation was found to reduce the ΔE across both the time points to a lesser level.

### Differential expression analysis of genes

In order to understand the mechanism of sprout inhibition due to radiation processing and associated quality retention during storage, comparative gene expression (transcriptomics) analysis of the radiation treated and control tubers were performed (Table S3A). Radiation treated and control potato tuber samples belonging to Santana, Kufri Frysona and Lady Rosetta varieties were collected in duplicate at 90 days of cold storage for RNA extraction followed by gene expression analysis. Day 90 of storage was selected because at this time point almost complete sprouting was observed in control tubers, whereas there was no sprouting in the irradiated samples.

**Figure 3:**
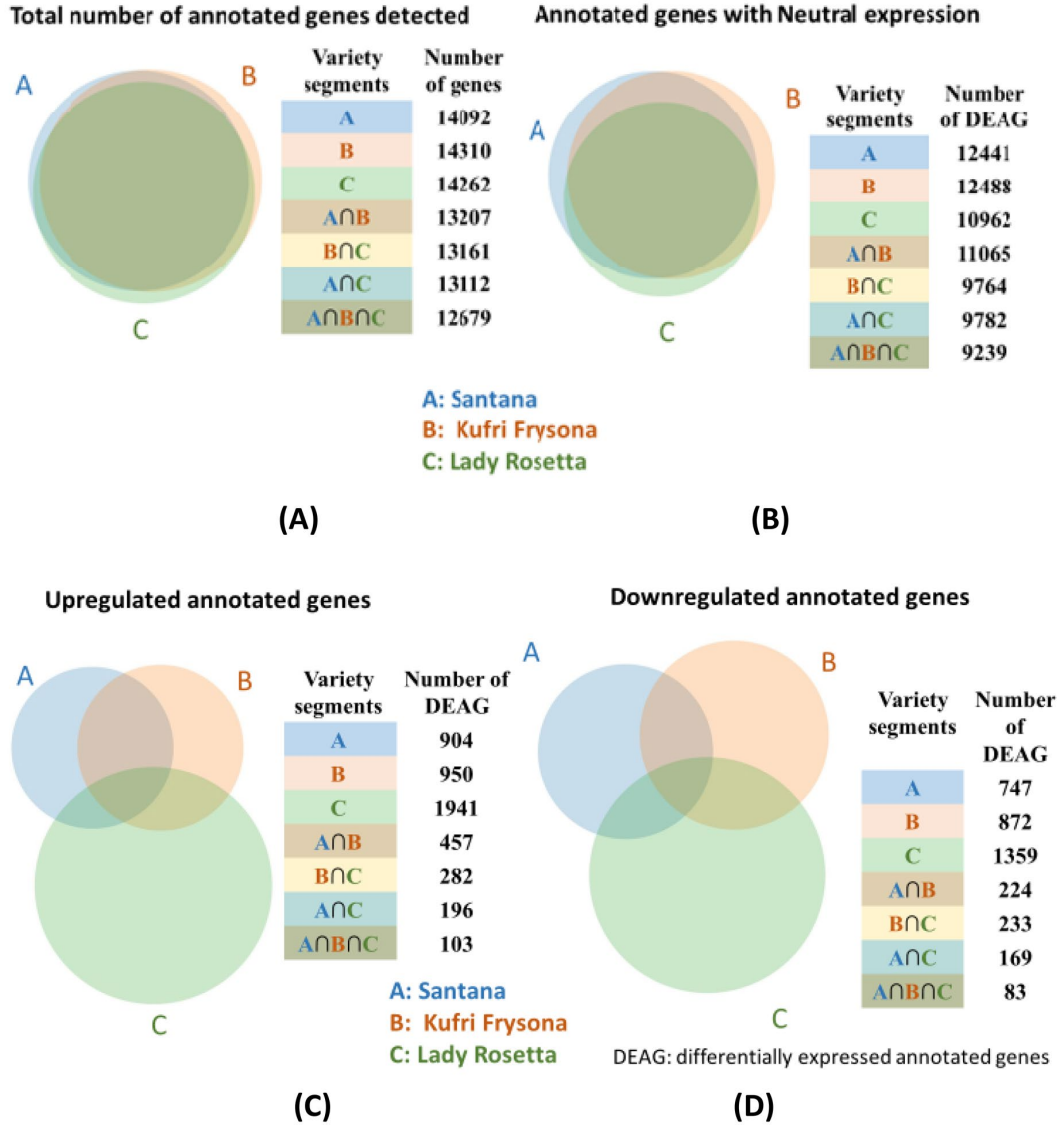
Venn diagram showing total annotated genes (**A**), annotated genes showing comparative neutral expression (**B**); as well as differentially expressed genes in terms of upregulation (**C**) and downregulation (**D**) in radiation treated tubers of the three potato varieties with respect to the control non-treated tubers of the respective varieties.

The summary of sequence reads after mapping onto the reference *Solanum tuberosum* (GCA_000226075.1) is presented in Table S3B and it exhibited high level (> 80%) of sequence alignment among all the three varieties. The comparative analysis was done based on annotated genes pertaining to carbohydrate metabolism that were identified in control and irradiated samples of each variety (Table S4). Fold changes (FC) in their expression upon radiation treatment were calculated for these genes in comparison to the respective control of that variety. A total of 14,341, 14,581 and 14,463 genes were expressed in Santana, Kufri Frysona and Lady Rosetta respectively, out of which 98 ± 0.2% had UniProt annotation assigned to them (Table S4). Inter-varietal comparison was based on 12,679 expressed genes having UniProt annotation which were detected in common in all the three varieties (Table S4, Fig. 3A). In these varieties; 12,441, 12,488 and 10,962 genes were found to show neutral expression level when compared to their respective control. Among these, 9239 genes were common to all three varieties (Fig. 3B). This reflects that on an average 84 ± 6% genes did not show any change in expression upon radiation treatment. Around 6.4, 6.6 and 13.6% annotated differentially expressed genes (DEGs) were found to be upregulated in these varieties among which 103 genes were identified in common (Fig. 3C). Similarly, 5.3, 6.1 and 9.5% DEGs were found to be down regulated in these varieties of which 83 genes were detected in all the three varieties (Fig. 3D).

Also, among annotated genes in Santana, 20 genes were upregulated more than 5 log2 FC and 33 genes were downregulated at less than − 5 log2 FC of which 4 were downregulated at less than − 10 log2 FC (Fig. S1). In Kufri Frysona, 15 genes were upregulated at 5 log2 FC and 13 genes were downregulated at − 5 log2 FC. In Lady Rosetta variety, 170 genes were upregulated at 5 log2 FC of which 13 were upregulated at ≥ 10 log2 FC. In this variety, 42 genes were downregulated at ≤ − 5 log2 FC and out of these 4 were downregulated at ≤ − 10 log2 FC.

### RNA-seq data indicated downregulation in expression of genes related to growth hormones and upregulation of genes related to dormancy hormones

**Figure 4:**
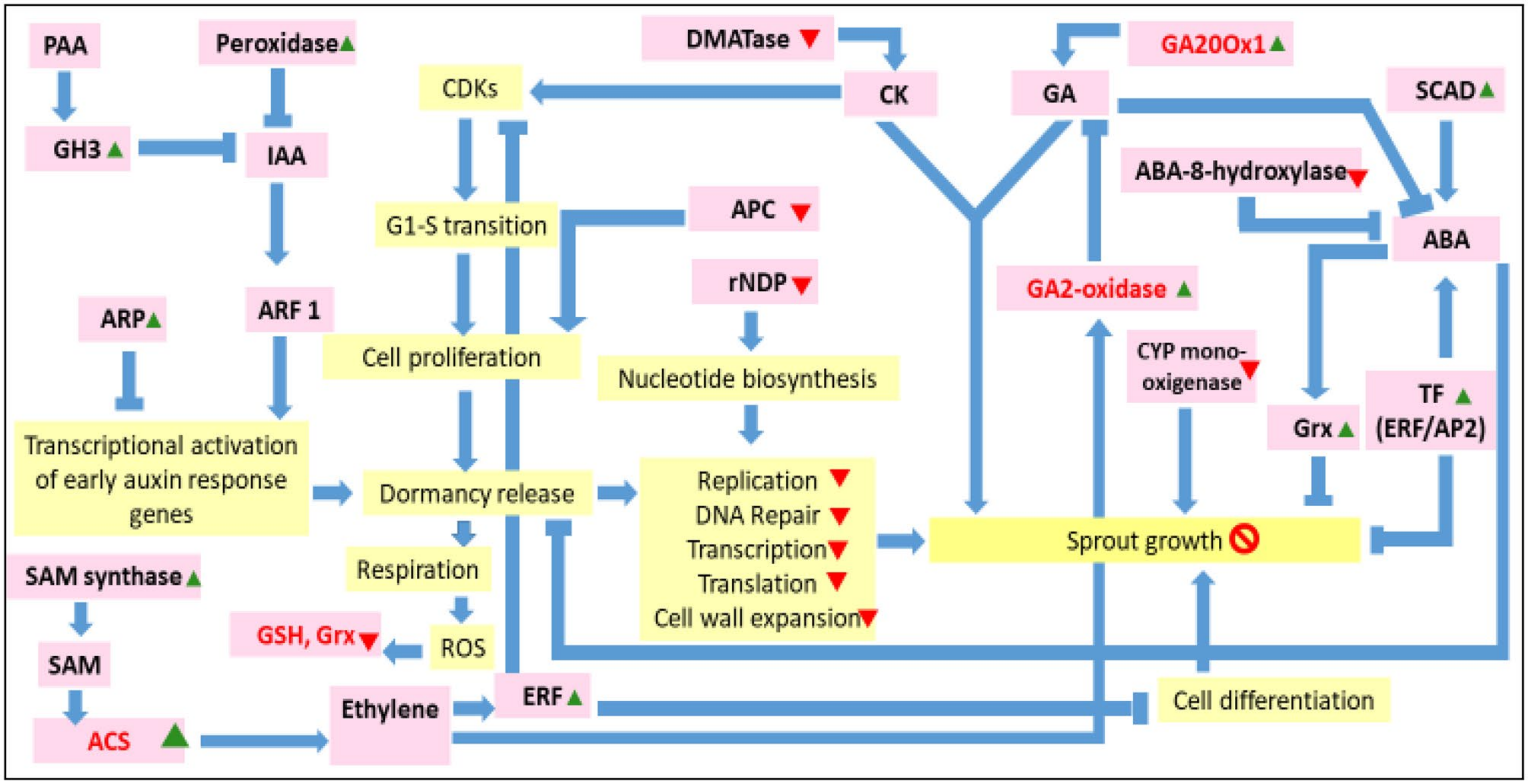
Proposed pathways depicting effect of low dose (60 Gy) gamma radiation on shelf life extension of potato tubers under storage achieved by sprouting inhibition and extend dormancy. In brief, high level of ABA due to downregulation of ABA 8′-hydroxylase and upregulation of short chain alcohol dehydrogenase (*SCAD*) gene may lead to upregulation of ABA regulated gene like Glutaredoxin (*Grx*) which has been reported to negatively affect germination and seedling growth. Rate limiting step of ethylene biosynthesis from SAM via ACC is catalyzed by ACC synthase (*ACS*) was upregulated which may lead to high ethylene level and prolonged dormancy in irradiated potatoes. Upregulation of *GH3* (*Gretchen Hagen3*) and auxin oxidase (*peroxidase*) as well as upregulation of repressor of early auxin response genes (*ARP*) may have also contributed to reduced auxin level and inhibition of sprout growth. Lower cytokinin due to down regulated *tRNA-DMATase* gene known to involve in its biosynthesis aided retained dormancy in irradiated potatoes. Furthermore, suppression of a number of genes related to cellular proliferation activities such as DNA synthesis, replication and cell division, translation and transcription machineries indicate reduction in metabolic activity and prolonged dormancy in radiation treated potato tubers. *PAA*: phenylacetic acid, *IAA*: indole acetic acid, *GH3:* Gretchen Hagen3, *ARP:* auxin repressed proteins, *ARF:* auxin response factors, *ERF*: ethylene response factor, *SAM:* S-adenosyl-l-methionine, *ACC*: 1-aminocyclopropane-1-carboxylic acid, *ACO*: 1-aminocyclopropane-1-carboxylic acid oxidase, *GSH*: glutathione, *CDK*: cyclin dependent kinase, *APC*: anaphase-promoting complex, *DMATase:* tRNA dimethyl allyl transferase, *CK:* cytokinin, *GA:* gibberellic acid, *ABA:* abscisic acid, *SCAD:* short chain alcohol dehydrogenase, *CYP:* cytochrome P450 mono-oxygenase, *rNDP:* ribonucleoside-diphosphate, *Grx*: glutaredoxin.

It is well known that dormancy break is guided by changes in the expression of several genes resulting in either enhancement in ‘growth and proliferation promoting factors’ and suppression of genes related to ‘sprouting inhibitory factors’. The differential profile of upregulated and down regulated genes in the sprouted (control) and un-sprouted (irradiated) tubers from the three varieties also reflected the regulatory roles of several sprouting related hormones such as ABA, GA, cytokinin and IAA. Phytohormones are signalling molecules, produced within plants at very low concentrations. ABA functions as growth inhibitor, auxin plays important role in cell elongation, root initiation and bud formation, cytokinin influences cell division and shoot formation, and gaseous hormone ethylene prevents cell elongation and helps in fruit ripening^54^. Role of plant hormones for sprout inhibition in radiation treated potatoes were quite evident from the comparative transcriptome analysis data of control and radiation processed potato varieties (Tables 1, 2, Fig. 4).

**Table 1:** Downregulated genes involved in dormancy/sprout inhibition in radiation treated potato tubers stored in cold for 90 days.

Gene names	UniProt entry name	Protein names	Functional category	Function	Fold change (downregulated)		
					Santana	Kufri Frysona	Lady Rosetta
PGSC0003DMG400028143	M1CQ28_SOLTU	Replication factor A component	Replication, repair and cell division	Replication, cell division	27.67	6.48	11.59
PGSC0003DMG400019692	M1BQQ1_SOLTU	ribonucleoside-diphosphate reductase complex (rNDPC)		Deoxyribonucleotide biosynthesis	2.47	2.88	2.68
PGSC0003DMG400016248	M1AQH4_SOLTU	Cell cycle switch 52A		Component of the anaphase promoting complex, cell cycle-regulated E3 ubiquitin-protein ligase complex that ensures unidirectional progression of cell cycle	2.26	3.30	3.23
PGSC0003DMG400019205	M1BNQ5_SOLTU	Uncharacterized protein		Nuclear division, regulation of DNA endoreduplication	806.55	86.44	489.40
PGSC0003DMG400016248	M1BC64_SOLTU	Chromatin assembly factor 1, subunit A (CAF-1A)		Part of CAF-1 complex required in postreplicative chromatin synthesis by facilitating nucleosome assembly through histone biosynthesis. Involved in cell division and double-strand break repair via homologous recombination	2.91	3.99	3.48
PGSC0003DMG401020221	M1BSS9_SOLTU	Sister chromatid cohesion 1 protein (SCC1)		Sister chromatid cohesion	5.55	2.52	2.87
PGSC0003DMG400013008	M1AZT2_SOLTU	Rad7		Nucleotide excision repair	4.01	4.81	2.60
PGSC0003DMG400020162	M1BSI1_SOLTU	Protein XRI1		Meiotic nuclear division, meiotic DNA repair	23.82	7.28	5.66
PGSC0003DMG401030070	M1CY68_SOLTU	Poly [ADP-ribose] polymerase (PARP)		Involved in the base excision repair (BER) pathway	23.54	8.42	5.40
PGSC0003DMG400012761	M1AYS8_SOLTU	Ss DNA-dependent ATP-dependent helicase,Ku P80 DNA helicase		Ku70:Ku80 complex for DNA repair	3.97	3.03	5.05
PGSC0003DMG400014226	M1B4G4_SOLTU	Forkhead-associated (FHA) domain-containing protein		Single/double-strand break repair	2.27	2.09	4.02
PGSC0003DMG401017663	M1BHU1_SOLTU	DNA-directed RNA polymerase subunit beta	Transcription	Transcription	2.41	5.93	3.26
PGSC0003DMG402017663	M1BHU2_SOLTU	DNA-directed RNA polymerase subunit beta		rpo B	3.04	9.05	2.27
PGSC0003DMG400017664	M1BHU1_SOLTU	DNA-directed RNA polymerase		Transcription	4.13	13.52	3.75
PGSC0003DMG400017664	M1BHU5_SOLTU	DNA-directed RNA polymerase			4.13	7.89	3.75
PGSC0003DMG400020296	M1BT57_SOLTU	DNA-directed RNA polymerase		rpo C2	3.86	10.40	2.62
PGSC0003DMG402029613	M1CW95_SOLTU	DNA-directed RNA polymerase		rpo C1	2.99	13.52	2.23
PGSC0003DMG400028306	M1CQV4_SOLTU	60S ribosomal protein L7a	Translation	Translation	2.18	2.21	2.91
PGSC0003DMG400019677	M1BQM9_SOLTU	Elongation factor 1-alpha (EF1A)		Promotes binding of aminoacyl-tRNA to the A-site of ribosomes during protein biosynthesis	11.64	41.40	9.74
PGSC0003DMG400029503	M1CVV8_SOLTU	Xyloglucan:xyloglucosyl transferase, Hydrolase (XTH)	Cell wall	Cell wall expansion, reconstruction, and re-modelling	6.50	3.93	21.79
PGSC0003DMG400000957	M0ZK83_SOLTU	tRNAdimethylallyltransferase (tRNA DMATase)	Phytohormo-ne	Zeatin biosynthesis	2.73	6.82	3.32
PGSC0003DMG400007972	M1ADR8_SOLTU	ABA 8′-hydroxylase CYP707A2		ABA acid 8′-hydroxylase activity	4.76	4.30	6.71
PGSC0003DMG400017314	M1BGG1_SOLTU	CYP72A57	Others	Heme binding monooxygenase, its downregulation results in sprout suppression	3.41	2.97	9.83
PGSC0003DMG400012669	M1AYE7_SOLTU	Cytochrome P450		Membrane bound heme binding monooxygenase	7.49	3.47	2.33

**Table 2:** Upregulated genes involved in dormancy/sprout inhibition in radiation treated potato tubers stored for 90 days.

Gene names	UniProt entry name	Protein names	Functional category	Function	Fold change (upregulated)		
					Santana	Kufri Frysona	Lady Rosetta
PGSC0003DMG400025282	M1CDC2_SOLTU	Apetala2/Ethylene Response Factors (AP2/ERF) domain-containing transcription factor	ABA metabolism	Positively regulate cold response, ethylene response and ABA response; negatively regulate development	31.32	9.60	3.96
PGSC0003DMG400036493	M1DCS9_SOLTU	AP2/ERF domain-containing transcription factor		Positively regulate cold response, ethylene response and ABA response; negatively regulate development	14.17	4.30	3.01
PGSC0003DMG400037250	M1DEB7_SOLTU	S-adenosylmethionine synthase (SbSAMS)		Biosynthesis of SAM, precursor of ethylene, SAM upregulates ABA and ethylene biosynthesis path genes	2.42	3.77	2.89
PGSC0003DMG400016744	M1BE67_SOLTU	Short chain alcohol dehydrogenase (SCAD)		Penultimate step of ABA biosynthesis, ABA helps embryo to survive desiccation during dormancy	17.30	9.86	107.27
PGSC0003DMG400024249	M1C8V8_SOLTU	Gibberellin 20-oxidase-1 (StGA20ox1)	GA metabolism	Key oxidase enzyme in the biosynthesis of gibberellin. StGA20ox1 controls stem elongation and tuber formation but has no role in dormancy in potato tuber	2.52	3.17	7.03
PGSC0003DMG402025083	M1CCJ9_SOLTU	Auxin oxidase (PRX: Peroxidase)	Auxin metabolism	Auxin catabolism	3.15	5.63	131.52
PGSC0003DMG400020139	M1BSE6_SOLTU	Auxin-responsive protein (ARP)		Transcriptional factors, repressors of early auxin response genes	59.30	6.55	20.87
PGSC0003DMG400006108	M1A6C5_SOLTU				4.67	2.59	15.67
PGSC0003DMG400019302	M1BP59_SOLTU				2.37	2.65	2.43
PGSC0003DMG400025856	M1CFP5_SOLTU	Auxin and ethylene responsive Gretchen Hagen 3 (GH3)		IAA homeostasis by encoding auxin conjugating enzymes	7.32	6.67	24.07
PGSC0003DMG400012186	M1AW72_SOLTU	1-amino-cyclopropane-1-carboxylate synthase (ACS)	Ethylene metabolism	Second and rate limiting step of ethylene biosynthesis from SAM via ACC	26.93	4.13	3.31
PGSC0003DMG400003530	M0ZVK4_SOLTU	Abscisic acid and environmental stress-inducible protein TAS14		Osmotic and dehydration stress tolerance	136.36	3.99	96.32
PGSC0003DMG400008248	M1AEV1_SOLTU	Wound-responsive AP2 like factor 2 (WRAF2)	Others	Adaptation during seed dormancy	16.90	4.30	3.01
PGSC0003DMG400008264	M1AEW8_SOLTU				34.93	9.89	8.36
PGSC0003DMG400008265	M1AEW9_SOLTU				26.06	5.40	2.43
PGSC0003DMG400023910	M1C7H6_SOLTU	Exocyst subunit Exo70 family protein (EXO70)		Exocyst complex, determinant of cellular polarity therefore promote growth. Overexpression inhibits growth of pollen tube	15.56	3.45	2.28
PGSC0003DMG400023909	M1C7H5_SOLTU				21.32	3.45	2.28

Phytohormone ABA confers induction and maintenance of tuber dormancy and ABA 8′-hydroxylase is one of the most important modulatory enzymes in ABA catabolism. It converts ABA to biologically inactive phaseic acid and dihydrophaseic acid^55^. This gene was found to be downregulated by 4.76, 4.30 and 6.71-fold in irradiated tubers of Santana, Kufri Frysona and Lady Rosetta, respectively. Suppression of this enzyme activity contributed in dormancy maintenance in the radiation treated potato tubers. ABA is synthesized from zeaxanthin in a multistep process where xanthoxin is produced as an intermediate. Xanthoxin is converted to abscisic aldehyde by short chain alcohol dehydrogenase (*SCAD*, *PGSC0003DMG400016744*), and finally ABA is synthesized by aldehyde oxidase^56^. A *SCAD* gene was upregulated in radiation treated tubers by 17.30, 9.86 and 107.27-fold in Santana, Kufri Frysona and Lady Rosetta varieties, respectively, compared to their respective non-irradiated control tubers. Besides, Glutaredoxin, an ABA regulated gene was found to be upregulated by 33.4, 2.3 and 7.1-fold in Santana, Kufri Frysona and Lady Rosetta varieties, respectively. This gene has been reported to negatively affect the germination and seedling growth^57^. This may be a key regulatory mechanism involved in radiation induced inactivation of sprouting by prolonging tuber dormancy and thereby associated shelf life extension. Here, enzyme involved in ethylene biosynthesis such as ACS (*ACC synthase*, *PGSC0003DMG400012186*) which is known to convert S-adenosyl-l-methionine (SAM) to 1-aminocyclopropane-1-carboxylic acid (ACC) has been found to be up regulated by 26.93, 4.13 and 3.31 fold in Santana, Kufri Frysona and Lady Rosetta, respectively. ACS catalyzed reaction is reported to be the rate limiting step in ethylene biosynthesis and its upregulation indicated higher production of ethylene in the radiation treated tubers. In general, increase in ethylene has been reported to be associated with dormancy maintenance in potato tubers^58^. Also, in other studies, exogenous application of ethylene has been reported to prevent sprouting in potatoes^59^.

Role of cytokinin in dormancy breakage has been emphasized in an earlier study^60^. Zeatin riboside is one of the major cytokines in potato tuber and has been shown to increase in tuber buds during termination of dormancy^61^. The transcriptome profile of Santana, Kufri Frysona and Lady Rosetta revealed 2.73, 6.82 and 3.32-fold decrease respectively in the level of a tRNA dimethyl allyl transferase (*tRNA-DMATase*, *PGSC0003DMG400000957*) known to be involved in the biosynthesis of zeatin. GA is known to be the classic dormancy terminator hormone in plant but presence of cytokinin is obligatory for GA dependent release of tuber dormancy. GA20 oxidase is a major enzyme in the biosynthetic pathway of GA, therefore an increase in its expression upon radiation treatment appears contradictory. But in a study involving StGA20ox1, a GA20 oxidase, it was shown that the enzyme was involved in tuber formation and stem elongation in potato rather than in sprouting^62^.

In current study, five auxin metabolism related genes were upregulated in all the three radiation processed varieties. Three of these genes are short lived repressor which act as inhibitor of transcriptional regulator of ARF (auxin response factor) resulting in negative regulation of early auxin response genes under low auxin concentration^63^. One of these AUX/IAA protein encoding genes (*ARP*, *PGSC0003DMG400020139*) was 60 and 20 times upregulated in irradiated Santana and Lady Rosetta and 6 times upregulated in Kufri Frysona. Aux/IAA repressor protein has been reported to be effective at low auxin concentration and it gets degraded when auxin concentration is high^64^. Auxin oxidase (*peroxidase*, *PGSC0003DMG402025083*) involved in its catabolism was found to be 3, 6 and 132 times upregulated in irradiated Santana, Kufri Frysona and Lady Rosetta varieties, respectively^65^. *GH3* (*Gretchen Hagen3*), a gene responsible for IAA homeostasis, encode for auxin conjugating enzymes was also upregulated 7–24 times lowers IAA availability causing reduction in sprout growth.

### Expression of genes other than phytohormones relevant to dormancy break

Other genes were also found to have contrasting expression profile in the control sprouted and radiation treated un-sprouted potatoes. Heme binding monooxygenase activity has been earlier reported to be associated with a large number of primary and secondary metabolite biosynthesis in plants, and their suppression have been cited to be indictor for inhibition of sprouting^66^. Exocyst subunit 70 was another commonly upregulated gene in radiation treated potato tubers. Role of such protein in context of tuber dormancy have not been studied yet, however a study on overexpression of exocyst 70 in *Arabidopsis* has showed retardation in the growth of pollen tube^67^.

Transcriptome analysis of irradiated potato tubers have indicated suppression of genes related to core cellular proliferation activities such as DNA synthesis, replication and cell division (Table 1). Five genes related to either cell division, nucleic acid biosynthesis and cell cycle progress [Replication factor A (*RRA*) component, neural proliferation, differentiation and control protein analog (*rNDPC*), Cell cycle switch *52A*, chromatin assembly factor (*CAF-1A*), sister chromatid cohesion protein (*SCC1*)] in all the three radiation treated varieties of potato were downregulated. Besides, down regulation of five DNA repair genes (*Rad7*, *XRI1*, *PARP*, *ss DNA helicase*, *Ku P80* and *Fork head-associated (FHA) domain-containing protein*) of which three are involved in strand break repair (*XRI1*, *ssDNA helicase* and* FHA domain containing protein*), two are involved in excision repair (*Rad7* and *PARP*) and one in homologous recombination repair (*Ku 80*) was observed. Also 2–13-fold suppression was noted in six genes of DNA-directed RNA polymerase related to cellular transcription and two genes (*RPL7A:PGSC0003DMG400029503* & *EF1APGSC0003DMG400019677*) involved in translation machinery were also downregulated. Therefore, the observed downregulation could be attributed to reduction in cell division and DNA biosynthesis in the radiation treated potato tubers indicating prolonged dormancy in the same. Moreover, a gene coding for Xyloglucan: xyloglucosyl transferase (*XTH*, *PGSC0003DMG400029503*) involved in cell wall biosynthesis and remodeling was also found to be 4–21 times downregulated in all the varieties.

### Endogenous phytohormone level in potato tubers

Endogenous phytohormone estimation indicated different levels depending upon varieties (Table S5). In Santana, Kufri Frysona and Lady Rosetta control potatoes, auxin (IAA) was 198.12 ± 5.6, 268.21 ± 19.7 and 59.61 ± 0.96 ng/g of fresh weight, respectively. In irradiated potatoes of these varieties, IAA level was found to be 139.97 ± 3.38, 85.39 ± 0.79 and 7.77 ± 0.12 ng/g, respectively. Thus, radiation treatment of potatoes was found to reduce IAA level by 30, 68 and 87% in these varieties, respectively. ABA content in control potato varieties was found to be 5.07 ± 0.33, 17.85 ± 0.99 and 14.9 ± 0.61 ng/g, whereas in the case of irradiated ones it was 12.94 ± 0.48, 21.5 ± 0.83 and 25.86 ± 1.23 ng/g, respectively. Hence, in the respective irradiated samples, 61, 17 and 42% higher ABA content was observed. GA3 levels in control potato varieties were 11.92 ± 0.7, 10.66 ± 0.34 and 22.34 ± 0.75 ng/g, however in irradiated potatoes it was found to be higher in Santana (nine fold) and Lady Rosetta (two fold), but lower in Kufri Frysona (three fold) as compared to respective control. Thus, findings indicated major role of radiation treatment in maintaining the differential levels of IAA and ABA in potatoes and their dormancy state. The critical roles of IAA and ABA phytohormones in potato tuber dormancy have also been emphasized earlier^58^.

### Radiation treatment led to retention of macronutrients and minerals during prolonged cold storage

The nutritional analysis data for the potato varieties during storage have been shown in Table S6. Energy content in the control and irradiated varieties initially ranged between 87 and 104 kcal/100 g. This was not found to change significantly in irradiated samples during storage of 240 days. In control tubers changes were not significant up to 90 days, however during further storage these potatoes were unacceptable due to extensive sprouting. Therefore, these control samples were considered unsuitable for consumption as well as for nutritional quality assessment. Similarly, total carbohydrate content was also not found to significantly change in the irradiated samples during storage of 240 days. Carbohydrates are the major contributor for the energy which undergoes starch to sugar conversion. Protein and fat content was quite less in potato varieties. Ash and potassium were in the range of 0.72–1.0 and 184–294 mg/100 g, respectively, in controls (0 day) which were not found to change significantly in the respective irradiated samples during 240 days of storage. Notably, in Kufri Frysona, potassium content was significantly high as the other two varieties.

### Molecular basis for variation in level of reducing sugars and vitamins in potato varieties

**Figure 5:**
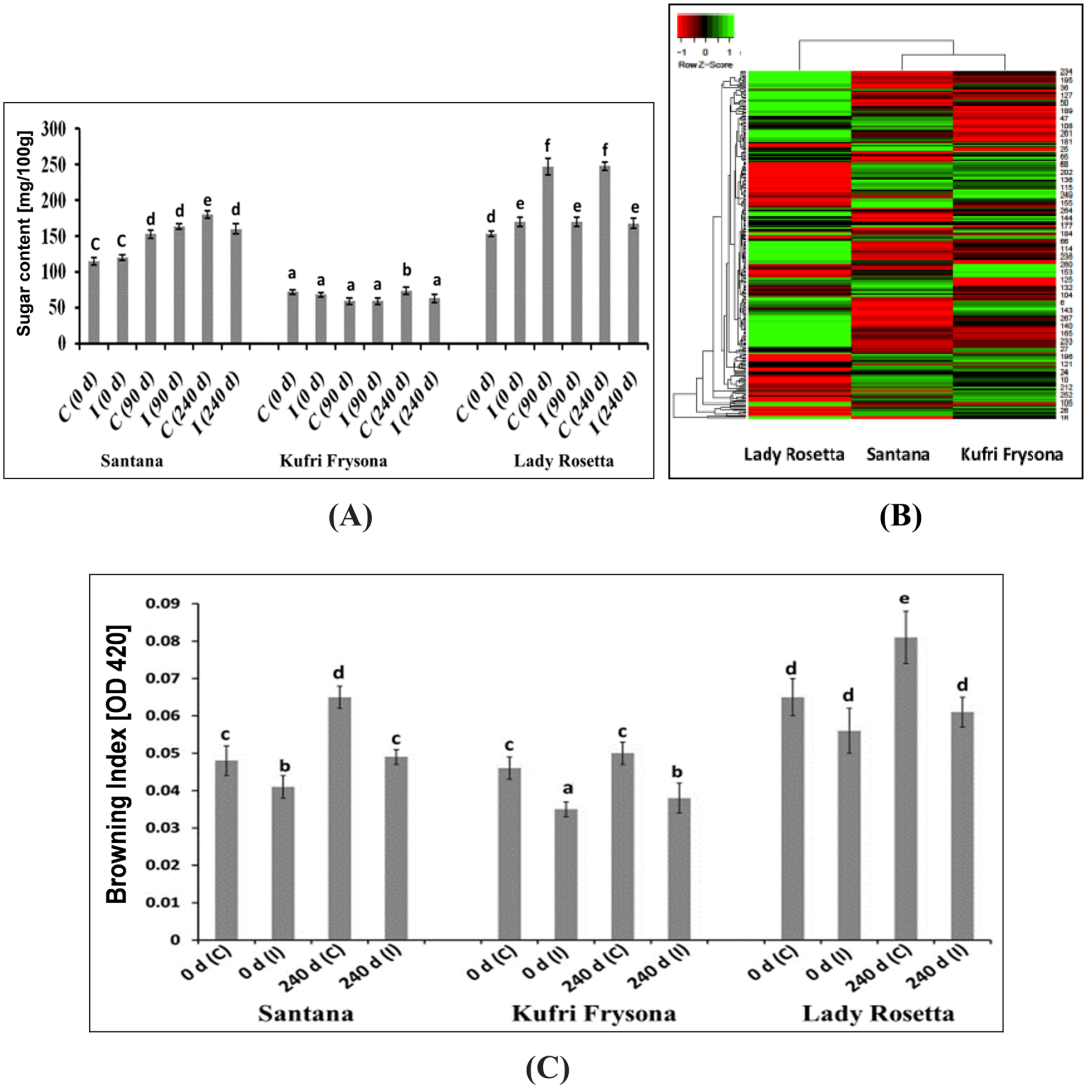
Reducing sugar content analysis (**A**), gene expression profile as represented by clustering of genes involved in carbohydrate, especially sugar metabolism (**B**) and Browning Index (BI) estimation (**C**) of non-irradiated control (C) and Irradiated (I) potato varieties during storage. Different letters on the top of the bars indicate statistically significant difference among means (p ≤ 0.05).

In the present study, all the three varieties contained less reducing sugar compared to previously reported varieties and thus well suitable for processing and chip-making^17^. However, during prolonged storage significant increase in reducing sugar was observed in control samples (Fig. 5A). The initial reducing sugar content of Santana and Kufri Frysona was between 68 and 115 mg/100 g (Fig. 5A). After 240 days of storage, the increase in the reducing sugar of control and irradiated samples of Kufri Frysona was 56 and 33%, respectively (Fig. 5A). Reducing sugar content in potatoes below 180 mg/100 g is considered desirable for chip production^68^. In Lady Rosetta variety, reducing sugar was 153 mg/100 g and within 90 days the increase in sugar was up to ~ 61% in control samples (Fig. 5A). However, in case of irradiated Lady Rosetta samples increase was negligible even after 240 days of storage (Fig. 5A). Besides, in the other two irradiated potato varieties too sugar build up was insignificant during storage (Fig. 5A). A total of 271 DEGs related to carbohydrate metabolism from all the three potato varieties were subjected to cluster analysis and it was found that expression of genes is more dissimilar in Lady Rosetta compared to Santana and Kufri Frysona, whereas the latter two were placed under the same cluster (Fig. 5B). In potato tubers, Kunitz-type inhibitors are a group of vacuolar serine protease inhibitors^69^ that act as inhibitors of invertase responsible for cold induced rise in reducing sugar content^70^. It was found to be 25.4, 1.9 and 11.9-fold upregulated in irradiated potato varieties of Santana, Kufri Frysona and Lady Rosetta, respectively.

The initial content of vitamin C in the control and irradiated samples belonging to all the three varieties was found to be almost similar (7.2–7.6 mg/100 g) (Table S6). However, it was found to reduce considerably during prolonged storage as have been reported in several earlier studies also^71^. On an average, the vitamin C content in control samples was found to reduce by 53 ± 6% after 90 days^72^. In irradiated samples, reduction in vitamin C content was found to be 46 ± 9 and 75 ± 5%, respectively, after 90 and 240 days from the initial value. This indicated reduction of vitamin C in both control and irradiated potatoes could possibly involve similar mechanism such as storage related oxidation^73^. However, both forms i.e., oxidized and reduced forms of vitamin C are available for human metabolism^73^. During transcriptome analysis major enzymes involved in ascorbic acid biosynthesis such as galactono-1,4-lactone dehydrogenase (GLDH) were at similar level in control and irradiated tuber samples (Table S7).

The initial content of vitamin B6 was found to be in the range of (0.34–1.89 mg/100 g) among the varieties and it was found to increase during storage (Table S6) as reported earlier in other studies^74^. After 90 days, vitamin B6 content was increased up to 123 and 182% in control and irradiated samples, respectively. Further during storage, vitamin B6 did not change significantly in both Santana and Kufri Frysona, whereas increased significantly in Lady Rosetta variety by 77%. In Santana and Kufri Frysona, vitamin B6 was higher by 3 and 23% in the irradiated samples compared to their respective control samples. Contrary to these, in irradiated Lady Rosetta tubers, ~ 25% decrease was noted (Table S6). Pyridoxine biosynthesis protein isoform A (M1CZB0_SOLTU) has earlier been predicted in plant system by drawing analogy from deoxy xylulose 5-phosphate dependent pathway in *E. coli*. This protein was found to be downregulated to a greater extent in Lady Rosetta (FC_Lady Rosetta_: − 2.8), moderately upregulated in Kufri Frysona (FC_Frysona_: 1.5) and very minor changes observed in Santana (FC_Santana_: 1.2). Its expression pattern seems to be quite related to the observed trend in the vitamin B6 content.

### Reduced Browning in irradiated cooked potato tubers

Browning index (BI) indicated that there was lesser darkening in the irradiated (fresh or stored samples) as compared to respective control (5C). This might be attributed to lower reducing sugars and consequently suppression of Maillard reactions in irradiated potatoes during cooking as discussed earlier. Further, low dose of radiation treatment of potatoes may have reduced the content of free soluble phenolics involved in the formation of ferrous-phenolic complex and its subsequent oxidation leading to After Cooking Darkening (ACD) effect. Shao et al. ^75^ has also reported decrease in certain free phenolics of the white rice samples upon radiation treatment at lower doses.

### Chip-making and sensory qualities well retained in irradiated tubers

Chip-making quality was initially acceptable for all the varieties and sensory rating was excellent (6). However, in control potato tuber samples sensory rating got affected adversely due to sprouting and further enhancement in reducing sugar content during storage. Therefore, after 90 days of storage, chips prepared from control tubers were not acceptable due to lack of crispiness and slight browning upon frying. Contrary to this in irradiated samples chip-making quality was found to be well retained and sensory rating was very good even after 240 days.

**Figure 6:**
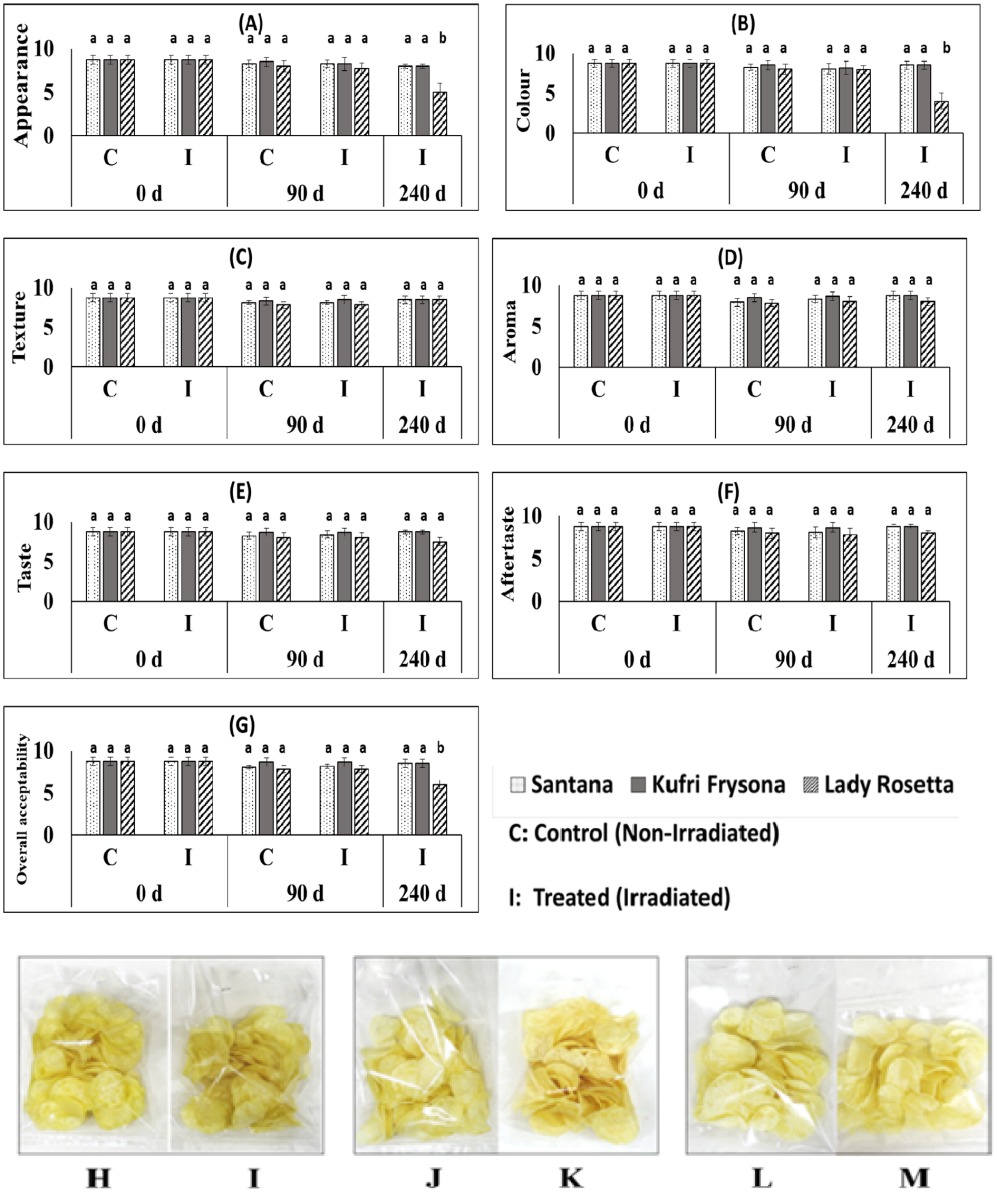
Sensory evaluation scores (**A** appearance, **B** colour, **C** texture, **D** aroma, **E** taste, **F** aftertaste, **G** overall acceptability) of chips prepared from treated and untreated potato varieties at different storage time. Chips prepared from untreated control (**H** Santana, **J** Kufri Frysona,** L** Lady Rosetta) and radiation treated (**I** Santana, **K** Kufri Frysona, **M** Lady Rosetta) potato after 90 days of storage. Different letters on the top of the bars indicate statistically significant difference among means (p ≤ 0.05).

### Correlation of differentially expressed relevant genes during transcriptome analysis and quality parameters

Correlation analysis showed that the decrease in auxin (IAA) content in irradiated potato varieties as compared to respective controls have high correlation (r = 0.78) with the expression of auxin oxidase (*peroxidase*) gene (Table S8). Further, expression of Aux/IAA repressor was also found to be highly correlated (r = 0.82) with the decrease in IAA content of these irradiated tubers (Table S8). Correlation of increase in the content of ABA was strong (r = 0.89) with Glutaredoxin gene expression, but it was having weak correlation (r = 0.15) with short chain alcohol dehydrogenase (*SCAD*) (Table S8). These results corroborated our findings and further indicated the role of IAA and ABA phytohormones as well as associated genes in the prolonged dormancy of irradiated potatoes. Gibberellin 20-oxidase-1 expression showed weak correlation (r = 0.09) with the increase in GA3 content (Table S8) and has been reported for lesser role in tuber dormancy^76^. Transcript levels of Kunitz-type invertase inhibitor of tubers from these varieties were strongly correlated (r = 0.81) with low reducing sugar content in irradiated tubers and thus plays important role in retaining its processing quality (Table S8). Expression profile of pyridoxine biosynthesis protein isoform A was found to be strongly correlated (r = 0.91) to the change in vitamin B6 levels in irradiated potatoes (Table S8).

### Predicted mitigation of post-harvest loss by deploying radiation technology and requirement of necessary infrastructure

India’s share with respect to area, production, annual processing and consumption has shown a linear increasing trend. Current share of potato farming in GDP is 2.86% (Fig. S2). Total cold storage capacity for various agricultural produce in India is ~ 37 MMT and 68% of this is used for storing potato tubers^77^. The study suggests that utilization of gamma irradiation may have a significant impact in reducing the post harvest loss (PHL at 16%) of 8.64 MMT of potatoes^78^ (Table S9). Besides, potato irradiation will be able to bring down projected PHL of 12.8 MMT of potatoes in 2050. With potatoes sharing about 3 billion USD in the Indian economy in 2020, commercial irradiation facilities for chip making potato variety may serve as a key driver to India’s potato export which is currently only ~ 1.7% of world’s total potato export^79–81^.

### Economic feasibility of potato tuber irradiation at commercial scale

Striking annual price variation was noted in major potato wholesale markets in all four zones in India (Fig. S3). When the price was analyzed all through the year, market price was found be highly fluctuating with 30% standard deviation (72.91 USD/MT) (Fig. S3)^82^. Considering cultivation cost (Table S9), a closer inspection reveals that from February to June, wholesale price stays very close to 200 USD/MT (horizontal dotted line; Fig. S3) and makes it difficult for farmers to earn good profit^82^. On the other hand, it appears that the price is very high from September to November. The cost for radiation (500 kCi capacity, Co-60 facility) of potatoes is 3.68% of the total cost (Table S9) which is ~ 6.5 times lesser compared to the cost of the seed tuber (~ 24% of the farming cost)^83^. Potato chips market in India showed 16.37% compound annual growth rate (CAGR) between 2016 and 2021 and more than 20% increase in sales value within a year in 2020–2021^84^. Thus, irradiation will provide significant commercial gains to potato farmers and other stakeholders involved in potato trade during harvest season. It may ensure availability of quality tubers meant for fast food and chip making industries during lean period and thus, radiation processing seems to be economically feasible and cost effective^84^ (Table S9).

### Plausible impact of potato farming and its irradiation on environment

Use of CIPC has health and environmental concerns^85^. Its usage has been banned in EU since January 2020. A single application of CIPC at 20 g/MT potato is required to prevent significant storage loss^86^. This application to total potato tuber produce may release ~ 67 kg of 3-CA (Table S9). Furthermore, impact of inclusion of potatoes as a staple diet instead of rice, wheat, and maize, in China has been shown to reduce greenhouse gas significantly and aided water conservation^44^. Average calorific values of rice, wheat and potato flours are quite comparable (366, 344 and 349 kcal/100 g)^87^. In potato farming, 65% less irrigation is required compared to rice, while the average yield of potato in India was found to be 7–8 times more than that of wheat and rice^26^. This indicates a denser calorific output from smaller agricultural land use, in case of potato (Table S9). India is on a mission to achieve carbon neutrality by 2070 and agriculture contributes 73% of total methane emissions^88^. As per the reports, diet based on potatoes was found to have 83 and 47% less global warming potential compared to rice and wheat, respectively^89^. Thus, potato farming and its extended preservation using ionizing radiation may aid in maintaining better environment and resource conservation.

## Summary

Current novel findings provide convincing molecular evidence affirming the efficacy of gamma radiation processing of potatoes at commercial scale for shelf-life extension with processing quality retention. Possible mechanism underlying sprout inhibition by ionizing radiation exposure seems to be attributed to differential regulation of genes pertaining to phytohormone biosynthesis and degradation. Genes related to ABA biosynthesis were upregulated whereas its catabolism was downregulated in irradiated potatoes. Converse expression was observed in the case of genes related to auxin in irradiated potatoes. Elevated expression of invertase inhibitor in irradiated potatoes may have an important role in retention of processing or chip-making quality. Further, quality retention in radiation exposed samples may also be related to reduction in the physiological changes occurring due to sprout inhibition and downregulation of genes involved in various metabolic pathways. The outcome of the current study provided evidence as well as opened an avenue for chemical sprout inhibitor free preservation of potato tubers by integrating radiation technology for extended periods.

### Supplementary Information

Supplementary Information.

## Data Availability

The data sets used and/or analyzed during the current study are available from the corresponding author on reasonable request. RNA sequencing data can be accessed at NCBI-SRA (National Center for Biotechnology Information—Sequence Read Archive) portal (BioProject ID: PRJNA1087157).
