# Supplementary material for: Differential gene expression in irradiated potato tubers contributed to sprout inhibition and quality retention during a commercial scale storage
Source: Sci Rep. 2024 Jun 12;14:13484. doi: 10.1038/s41598-024-58949-0 (PMC11169491; doi:10.1038/s41598-024-58949-0)
Supplement: Supplementary file 1 — Supplementary Information. [file 41598_2024_58949_MOESM1_ESM.doc]

**Supplementary Material**

**Table S1** Sprouting^*^ in non-irradiated control potato tubers of differed varieties

| **Varieties** | **% Sprouting**  **after 45 d** | **% Sprouting** | **Number of Sprout(s) per tuber** | **Sprout Length** |
| --- | --- | --- | --- | --- |
|  |  | **90 d** | | |
| Santana | 56±8^a^ | 100^a^ | 2±1^a^ | 4.9±1.8^a^ |
| Kufri Frysona | 50±7^a^ | 100^a^ | 2.1±1.1^a^ | 3.7±0.8^a^ |
| Lady Rosetta | 65±9^a^ | 100^a^ | 1.6±0.8^a^ | 5.9±0.8^b^ |

*No sprouting was observed in irradiated potato tubers

Note: Different letters rowwise (a-b) indicate signiﬁcant differences among means (P ≤ 0.05)

**Table S2A** Summary of colour estimation data obtained from radiation treated and non-irradiated control tubers at different storage period

| **Sample** | **L*** | **a*** | **b*** | **C*** | **H*** |
| --- | --- | --- | --- | --- | --- |
| Day 0, Control A# | 72.39±3.40^a^ | 0.45±0.15^d^ | 28.60±2.04^b^ | 28.60±2.04^b^ | 89.08±0.28^a^ |
| Day 0, Irradiated A | 64.46±2.29^b^ | 0.73±0.19^c^ | 25.82±2.33^b^ | 25.83±2.33^b^ | 88.35±0.33^a^ |
| Day 0, Control B# | 69.82±3.66^a^ | 0.46±0.25^d^ | 16.44±1.50^f^ | 16.45±1.50^e^ | 88.39±0.84^a^ |
| Day 0, Irradiated B | 71.72±3.69^a^ | 0.46±0.09^d^ | 22.20±5.11^c^ | 22.21±5.10^c^ | 88.71±0.43^a^ |
| Day 0, Control C# | 67.22±2.45^a^ | 0.61±0.06^c^ | 18.72±0.91^e^ | 18.73±0.91^d^ | 88.11±0.13^b^ |
| Day 0, Irradiated C | 68.90±1.85^a^ | 0.62±0.17^c^ | 20.29±0.61^d^ | 20.30±0.62^c^ | 88.24±0.43^b^ |
| Day 90, Control A | 58.22±2.49^c^ | 8.80±0.88 ^b^ | 32.80±2.34^a^ | 33.97±2.32^a^ | 74.91±1.61^c^ |
| Day 90, Irradiated A | 58.56±1.75^c^ | 9.19±0.74 ^b^ | 30.43±1.30^a^ | 31.80±1.37^a^ | 73.18±1.08^c^ |
| Day 90, Control B | 58.71±2.06^c^ | 9.52±0.53 ^a^ | 33.80±3.54^a^ | 35.12±3.53^a^ | 74.16±1.01^c^ |
| Day 90, Irradiated B | 58.76±2.40^c^ | 9.46±0.64 ^b^ | 32.08±3.05^a^ | 33.45±3.06^a^ | 73.48±1.09^c^ |
| Day 90, Control C | 52.82±2.26^d^ | 10.81±2.09^a^ | 24.59±2.12^c^ | 26.90±2.59^b^ | 66.40±3.12^e^ |
| Day 90, Irradiated C | 55.33±1.61^d^ | 9.36±0.71^b^ | 23.07±1.31^c^ | 24.90±1.42^c^ | 67.89±1.05^e^ |
| Day 240, Control A | 49.32±2.35^e^ | 10.53±0.66^a^ | 31.81±1.60^a^ | 33.51±1.53^a^ | 71.63±1.38^d^ |
| Day 240, Irradiated A | 57.07±2.64^c^ | 8.33±1.04^b^ | 33.61±1.14^a^ | 34.64±1.23^a^ | 76.06±1.55^c^ |
| Day 240, Control B | 57.42±2.64^c^ | 8.65±1.75^b^ | 32.00±4.33^a^ | 33.20±4.19^a^ | 74.64±3.47^c^ |
| Day 240, Irradiated B | 55.92±0.92^c^ | 10.15±0.71^a^ | 32.14±2.14^a^ | 33.70±2.19^a^ | 72.44±0.90^d^ |
| Day 240, Control C | 48.37±2.29^e^ | 11.68±1.17^a^ | 25.01±1.18^c^ | 27.61±1.50^b^ | 64.99±1.48^e^ |
| Day 240, Irradiated C | 52.48±2.31^d^ | 10.54±0.83^a^ | 24.17±1.34^c^ | 26.37±1.52^b^ | 66.44±0.91^e^ |

L*, a*, b*, C* and H* acronyms correspond to lightness, red (+) / green (-), yellow (+) / blue (-), chroma and hue, respectively. A#, B#, C#: Correspond to potato cultivars, Santana, Kufri Frysona and Lady Rosetta, respectively. Control: Non Irradiated control potato tubers; Irradiated: Radiation treated potato tubers. Superscript a-f denotes significant difference among the means across the column at P ≤ 0.05.

**Table S2B** Estimation of change in colour (∆E) of irradiated and non-irradiated control potato tubers at different storage period

| **Sample** | **∆E** | **Sample** | **∆E** | |
| --- | --- | --- | --- | --- |
| **Difference arising due to storage** | | **Difference arising due to radiation** | | |
| Control A# (d*0) vs Control A (d 90) | 16.97 | Control A (d 0) vs Irradiated A (d 0) | | 8.41 |
| Control A (d 0) vs Control A (d 240) | 25.38 | Control B (d 0) vs Irradiated B (d 0) | | 6.07 |
| Control B# (d 0) vs Control B (d 90) | 22.51 | Control C (d 0) vs Irradiated C (d 0) | | 2.30 |
| Control B (d 0) vs Control B (d 240) | 21.52 | - | |  |
| Control C# (d 0) vs Control C (d 90) | 18.60 | Control A (d 90) vs Irradiated A (d 90) | | 2.42 |
| Control C (d 0) vs Control C (d 240) | 22.75 | Control B (d 90) vs Irradiated B (d 90) | | 1.72 |
| - |  | Control C (d 90) vs Irradiated C (d 90) | | 3.27 |
| Irradiated A (d 0) vs Irradiated A (d 90) | 11.29 | - | |  |
| Irradiated A (d 0) vs Irradiated A (d 240) | 13.16 | Control A (d 240) vs Irradiated A (d 240) | | 8.25 |
| Irradiated B (d 0) vs Irradiated B (d 90) | 18.61 | Control B (d 240) vs Irradiated B (d 240) | | 2.12 |
| Irradiated B (d 0) vs Irradiated B (d 240) | 21.02 | Control C (d 240) vs Irradiated C (d 240) | | 4.34 |
| Irradiated C (d 0) vs Irradiated C (d 90) | 16.38 | - | |  |
| Irradiated C (d 0) vs Irradiated C (d 240) | 19.57 | - |  | |

A#, B#, C#: Correspond to potato cultivars Santana, Kufri Frysona and Lady Rosetta respectively; *D indicated days.

Control: non irradiated control potato tubers; Irradiated: radiation treated potato tubers.

**Table S3A** RNA sequence quality control data

| **Treatment** | **Cultivar** | **Replicate** | **Q20 (%)** | **Q30 (%)** | **GC**  **(%)** | **Q20 (%)** | **Q30 (%)** | **GC**  **(%)** | |
| --- | --- | --- | --- | --- | --- | --- | --- | --- | --- |
|  |  |  | Before Filtering | | | After Filtering | | |  |
| Control | Santana | 1 | 97.6 | 93.4 | 44.7 | 98.6 | 94.9 | 44.7 |  |
|  |  | 2 | 96.8 | 92.4 | 44.7 | 98.5 | 94.9 | 44.6 |  |
|  | Kufri Frysona | 1 | 97.4 | 93.1 | 45.1 | 98.5 | 94.9 | 45.1 |  |
|  |  | 2 | 96.8 | 92.4 | 43.5 | 98.5 | 94.8 | 43.3 |  |
|  | Lady Rosetta | 1 | 97.4 | 93.1 | 42.5 | 98.5 | 94.6 | 42.4 |  |
|  |  | 2 | 96.4 | 91.9 | 43.8 | 98.5 | 94.9 | 43.5 |  |
| Irradiated | Santana | 1 | 97.1 | 92.8 | 43.4 | 98.6 | 95 | 42.9 |  |
|  |  | 2 | 97.1 | 92.8 | 43 | 98.5 | 94.8 | 43.2 |  |
|  | Kufri Frysona | 1 | 97 | 92.7 | 45.1 | 98.6 | 95 | 44.6 |  |
|  |  | 2 | 96.6 | 92.2 | 43.4 | 98.6 | 95.1 | 42.6 |  |
|  | Lady Rosetta | 1 | 96.8 | 92.6 | 50.1 | 98.6 | 95.1 | 50.4 |  |
|  |  | 2 | 97 | 92.6 | 43.5 | 98.5 | 94.8 | 43.2 |  |

**Table S3B** Summary of sequence read data after transcriptome mapping on reference genome of *Solanum tuberosum* (GCA_000226075.1)

| **Test Set** | **Cultivar** | **Total Reads** | **Paired End Aligned Reads** | **Paired End Unaligned Reads** | **Single End Aligned Reads** | **Single- End Unaligned Reads** | **Overall alignment%** |
| --- | --- | --- | --- | --- | --- | --- | --- |
| Control | Santana | 26454094±356468 | 21807871±160570 | 4646224±195897 | 3730854±488540 | 5561593±880335 | 89.5±1.5 |
|  | Kufri Frysona | 25803983±2877102 | 21201060±4144966 | 4602923±1267863 | 3169221±156008 | 6036626±2379719 | 88.0±6 |
|  | Lady Rosetta | 15930999.5±11166557 | 12718218±8887826 | 3212782±2278730 | 2516218±1745517 | 3909347±2811943 | 87.8±0.3 |
| Irradiated | Santana | 30605473±8371632 | 25082869±6498950 | 5522604±1872681 | 4841186±2263810 | 6204022±1481552 | 89.8±0.4 |
|  | Kufri Frysona | 18424175±6932049 | 14921292±5301274 | 3502883±1630774 | 3286107±1701700 | 3719659±1559849 | 90.0±0.5 |
|  | Lady Rosetta | 21031524±7192007 | 17946282±7228944 | 3085242±36936 | 2770961±47862 | 3399523±121735 | 91.4±3.2 |

**Table S4** Categorization of total annotated genes and genes involved in carbohydrate metabolism into up-regulated, down-regulated and neutral groups in three potato cultivars

|  | **Santana**  **(Cultivar A)** | **Kufri Frysona**  **(Cultivar B)** | **Lady Rosetta (Cultivar C)** | **Common to A and B** | **Common to B and C** | **Common to A and C** | **Common to A, B and C** | **Only in A** | **Only in B** | **Only in C** |
| --- | --- | --- | --- | --- | --- | --- | --- | --- | --- | --- |
| Total DEG | 14341 | 14581 | 14463 | - | - | - | - | - | - | - |
| Total DEGs considered for further analysis | 14092 | 14310 | 14262 | 13207 | 13161 | 13112 | 12679 | 452 | 621 | 668 |
| Total Up-regulated | 904 | 950 | 1941 | 457 | 282 | 196 | 103 | 354 | 314 | 1566 |
| Total Down-regulated | 747 | 872 | 1359 | 224 | 233 | 169 | 83 | 437 | 498 | 1040 |
| Total Neutral | 12441 | 12488 | 10962 | 11065 | 9764 | 9782 | 9239 | 833 | 898 | 655 |
| Carbohydrate metabolism | 271 | 254 | 250 | 254 | 239 | 250 | 239 | 32 | 15 | 11 |
| Carbohydrate metabolism up-regulated | 51 | 51 | 80 | 16 | 9 | 3 | 5 | 10 | 4 | 18 |
| Carbohydrate metabolism Down-regulated | 53 | 45 | 62 | 3 | 4 | 3 | 3 | 13 | 7 | 15 |
| Carbohydrate metabolism neutral | 167 | 158 | 108 | 59 | 18 | 15 | 75 | 35 | 30 | 4 |

**Table S5** Estimation of phytohormones in irradiated and non-irradiated control potato tubers at 90 days.

| **Samples** | **Abundance in tuber (ng. g-1 of F.W.)** | | |
| --- | --- | --- | --- |
|  | **IAA** | **ABA** | **GA3** |
| Control Santana tubers | 198.12±5.6^a^ | 5.07±0.33^a^ | 11.92±0.7^a^ |
| Irradiated Santana tubers | 139.97±3.38^b^ | 12.94±0.48^b^ | 116.54±0.68^b^ |
| Control Kufri Frysona tubers | 268.21±19.7^c^ | 17.85±0.99^c^ | 10.66±0.34^c^ |
| Irradiated Kufri Frysona tubers | 85.39±0.79^d^ | 21.5±0.83^d^ | 3.77±0.09^d^ |
| Control Lady Rosetta tubers | 59.61±0.96^e^ | 14.9±0.61^e^ | 22.34±0.75^e^ |
| Irradiated Lady Rosetta tubers | 7.77±0.12^f^ | 25.86±1.23^f^ | 48.38±1.3^f^ |

Note: Different letters rowwise (a-f) indicate signiﬁcant differences among means (P ≤ 0.05)

**Table S6 Nutritional analysis of control and radiation processed potato^#^ during storage**

| **Parameters** | **Santana** | | | | | |
| --- | --- | --- | --- | --- | --- | --- |
|  | **Control** | | | **Irradiated** | | |
|  | **0 d** | **90 d** | **240 d** | **0 d** | **90 d** | **240 d** |
| Energy  (k Cal/100 g) | 95±7^a^ | 109±16^a^ | - | 87±5^a^ | 112±17^a^ | 105±11^a^ |
| Carbohydrate (g/100 g) | 19.8±3^a^ | 25.4±5^a^ | - | 18.8±4^a^ | 26.2±4^a^ | 23.5±3^a^ |
| Protein  (g/100 g) | 3.9±0.4^a^ | 1.8±0.6^b^ | - | 2.9±0.5^a^ | 1.9±0.3^b^ | 1.6±0.3^b^ |
| Fat  (g/100 g) | 0.04±0.01^a^ | 0.04±0.00^a^ | - | 0.06±0.02^a^ | 0.03±0.01^a^ | 0.02±0.01^a^ |
| Vitamin C  (mg/100 g) | 7.4±0.8b^a^ | 3.9±0.6^b^ | - | 7.3±0.4^a^ | 3.38±0.5^b^ | 2.27±0.3^b^ |
| Vitamin B6  (mg/100 g) | 1.13±0.3 ^a^ | 1.93±0.3^b^ | - | 1.89±0.4 ^a^ | 1.99±0.6 ^a^ | 1.92±0.4 ^a^ |
| Ash  (g/100 g) | 0.87±0.05 ^a^ | 0.86±0.04^a^ | - | 0.72±0.09^a^ | 0.98±0.08^a^ | 0.81±0.05^a^ |
| Potassium  (mg/100 g) | 204.0±18^a^ | 169.6±14^b^ | - | 189.9±13^a^ | 177.6±15^b^ | 176±12^b^ |
|  | **Kufri Frysona** | | | | | |
|  | **Control** | | | **Irradiated** | | |
|  | **0 d** | **90 d** | **240 d** | **0 d** | **90 d** | **240 d** |
| Energy  (k Cal/100 g) | 104±5^a^ | 114±6^a^ | - | 101±8^a^ | 115±6 ^a^ | 107±4.2 ^a^ |
| Carbohydrate (g/100 g) | 22.4±5 ^a^ | 26.8±4 ^a^ | - | 22.75±4 ^a^ | 27.2±3 ^a^ | 24.6±0.9 ^a^ |
| Protein  (g/100 g) | 3.5±0.5^b^ | 1.5±0.6^a^ | - | 2.5±0.6 ^a^ | 1.6±0.4^b^ | 1.7±0.1^b^ |
| Fat  (g/100 g) | 0.06±0.02 ^a^ | 0.01±0.00^b^ | - | 0.03±0.01^a^ | 0.01±0.00^b^ | 0.02±0.01^b^ |
| Vitamin C  (mg/100 g) | 7.6±0.6^a^ | 3.5±0.4^b^ | - | 7.5±0.7 ^a^ | 4.8±0.6^b^ | 1.63±0.6^c^ |
| Vitamin B6  (mg/100 g) | 0.35±0.04^a^ | 0.78±0.05^b^ | - | 0.34±0.03^a^ | 0.96±0.05^c^ | 0.95±0.04^c^ |
| Ash  (g/100 g) | 1.0±0.15^a^ | 1.23±0.04^a^ | - | 1.06±0.13^a^ | 1.28±0.04^a^ | 1.09±0.14^a^ |
| Potassium  (mg/100 g) | 294±12^a^ | 233.3±17^b^ | - | 282.6±16^a^ | 239.8±14^b^ | 269.5±16^b^ |
|  | **Lady Rosetta** | | | | | |
|  | **Control** | | | **Irradiated** | | |
|  | **0 d** | **90 d** | **240 d** | **0 d** | **90 d** | **240 d** |
| Energy  (k Cal/100 g) | 93±4^a^ | 102±6^a^ | - | 86±5^a^ | 103±4^a^ | 107±8^a^ |
| Carbohydrate (g/100 g) | 19.3±6^a^ | 24.0±7^a^ | - | 18.7±5^a^ | 24.9±6^a^ | 25.1±4^a^ |
| Protein  (g/100 g) | 3.9±0.8^a^ | 1.4±0.3^b^ | - | 5.2±0.6^a^ | 1.6±0.2^b^ | 1.4±0.1^b^ |
| Fat  (g/100 g) | 0.04±0.02 ^a^ | 0.02±0.01^a^ | - | 0.03±0.01^a^ | 0.02±0.01^a^ | 0.02±0.01^a^ |
| Vitamin C  (mg/100 g) | 7.5±0.7^a^ | 3.1±0.5^b^ | - | 7.4±0.4^a^ | 3.8±0.5^b^ | 1.6±0.2^c^ |
| Vitamin B6  (mg/100 g) | 0.75±0.03^a^ | 1.39±0.04^b^ | - | 0.82±0.05^a^ | 1.04±0.07^b^ | 1.84±0.04^c^ |
| Ash  (g/100 g) | 0.87±0.14^a^ | 1.04±0.15^a^ | - | 0.76±0.11^a^ | 1.05±0.17^a^ | 1.16±0.11^a^ |
| Potassium  (mg/100 g) | 184±17^a^ | 222±11^a^ | - | 218±14^a^ | 183±19^a^ | 231±21^a^ |

Note: Different letters columnwise (a-c) indicate signiﬁcant differences among means (P ≤ 0.05);

‘-‘ Samples unsuitable for consumption and nutritional quality assessment.

**Table S7** Expression profile of some genes (known to be involved in ascorbic acid biosynthesis in plants) in control and radiation treated potato belonging to cultivar A (Santana), B (Kufri Frysona) and C (Lady Rosetta)

| **Entry Name (Uniport)** | **Protein Names** | **Gene Names** | **Fold Change A** | **P value (A)** | **Fold Change B** | **P value (B)** | **Fold Change C** | **P value (C)** |
| --- | --- | --- | --- | --- | --- | --- | --- | --- |
| M1AZE3_SOLTU | Glucose-6-phosphate isomerase (EC 5.3.1.9) | 102585856 | -1.01 | 0.97 | 1.17 | 0.64 | 1.83 | 0.02 |
| M1CHZ5_SOLTU | Mannose-6-phosphate isomerase (EC 5.3.1.8) |  | -1.05 | 0.87 | -1.3 | 0.57 | -1.11 | 0.82 |
| M1ANX7_SOLTU | Mannose-6-phosphate isomerase (EC 5.3.1.8) | 102602821 | ND |  | -1.38 | 0.51 | -1.03 | 0.98 |
| M1AUP3_SOLTU | Mannose-6-phosphate isomerase (EC 5.3.1.8) | 102580489 | 0.88 | 0.71 | 1.42 | 0.29 | 1.95 | 0.04 |
| M1BYE0_SOLTU | Phosphomannomutase (EC 5.4.2.8) | 102589310 | -1.11 | 0.68 | 1.20 | 0.58 | -1.15 | 0.67 |
| M1A512_SOLTU | Phosphomannomutase (EC 5.4.2.8) |  | 1.34 | 0.36 | 1.3 | 0.43 | -1.07 | 0.86 |
| M1A6A6_SOLTU | GDP-mannose 3',5'-epimerase | 102582256 | 1.01 | 0.96 | 1.28 | 0.55 | -1.91 | 0.23 |
| A0A0B4J3K8_SOLTU | GDP-mannose 3',5'-epimerase | 102577923 | -1.08 | 0.8 | 1.27 | 0.55 | 3.10 | 0.01 |
| M0ZTX3_SOLTU | L-galactose dehydrogenase | 102599558 | -1.45 | 0.18 | 1.25 | 0.55 | -1.62 | 0.19 |
| M1AEF2_SOLTU | L-galactono-1,4-lactone dehydrogenase protein | 102577860 | -1.25 | 0.4 | -1.06 | 0.90 | 1.17 | 0.54 |

**Table S8** Correlation of Differentially Expressed Gene (DEG) in irradiated tubers and quality related constituents from different varieties with respect to control

| **DEG** |  | **Quality related constituents** | **Correlation Value (r)** |
| --- | --- | --- | --- |
| Upregulation of Auxin oxidase (peroxidase) gene |  | Decrease in Auxin (IAA) content | 0.78 |
| Upregulation of Aux/IAA repressor protein |  | Decrease in Auxin (IAA) content | 0.82 |
| Upregulation of Glutaredoxin gene |  | Increase in Abscisic Acid (ABA) content | 0.89 |
| Upregulation of Short chain alcohol dehydrogenase (SCAD) |  | Increase in Abscisic Acid (ABA) content | 0.15 |
| Upregulation of Gibberellin 20-oxidase-1 |  | Increase in GA3 content | 0.09 |
| Upregulation of Kunitz-type invertase inhibitor |  | Decrease in Reducing Sugars | 0.81 |
| Upregulation of pyridoxine biosynthesis protein isoform A |  | Increase in B6 Level | 0.91 |

**Table S9** Economic sustainability and environmental impact of commercial radiation processing of potato at national scale in India

| Cost (USD/MT) | | | | | | | | | | | | | | |
| --- | --- | --- | --- | --- | --- | --- | --- | --- | --- | --- | --- | --- | --- | --- |
| Farming | HSPLT† | | Storage | | Total | | Radiation †† | Over all | Mean national wholesale rate ‡ | Contribution of radiation | Chip manufacturing‡‡ | | Potato chips sales ‡‡‡ | |
| 157.2 | 24.4 | | 28 | | 210 | 8.04 | | 217.64 | 239.73±72.91 | 3.68% | 334.40 | | 487.29 | |
| Impact of CIPC | | | | | | | | | | | | | | |
| CIPC required for potato♦ | | | | | | Storage♦♦♦ | | 3-CA generation♦♦♦♦ | | | | | | |
| 20 g/MT | | | 1084.6 MT♦♦ | | | 1538 L/MT | | 0.4 µg/L of storage | | 615.2 µg/MT | | | 67 kG ## | |
| Yield (MT/ha)  | | | | | | Irrigation (mm) | | | | Carbon footprint of food product (kg CO2 eq. MT^−1^) | | | | |
| Rice | | Wheat | | Potato | | Rice | | Wheat | Potato | Rice | | Wheat | | Potato |
| 3 | | 3.5 | | 24 | | 1700±1131 | | 550±141 | 600±141 | 786 | | 250.6 | | 132 |

† HSPLT: harvesting, sorting, packaging, loading and transport cost, †† Quarterly average of annual operational cost on no loss-no profit basis considering full time (20 h) engagement in potato irradiation.

‡ Calculated by taking annual average of four major wholesale markets in four zones as depicted in Fig S3, ‡‡‡ (Murray, 2005)

♦ Calculations based on reported requirement for 30% storage loss

♦♦For total harvest, ♦♦♦Assuming 100% occupancy, ♦♦♦♦ (Park and Duncan, 2009; Vijay et. al., 2018)

 Calculated from Economic Survey 2022-2023, Statistical Appendix. Directorate of Economics and Statistics, DAFW. Table 1.17: Yield Per Hectare of Major Crops.


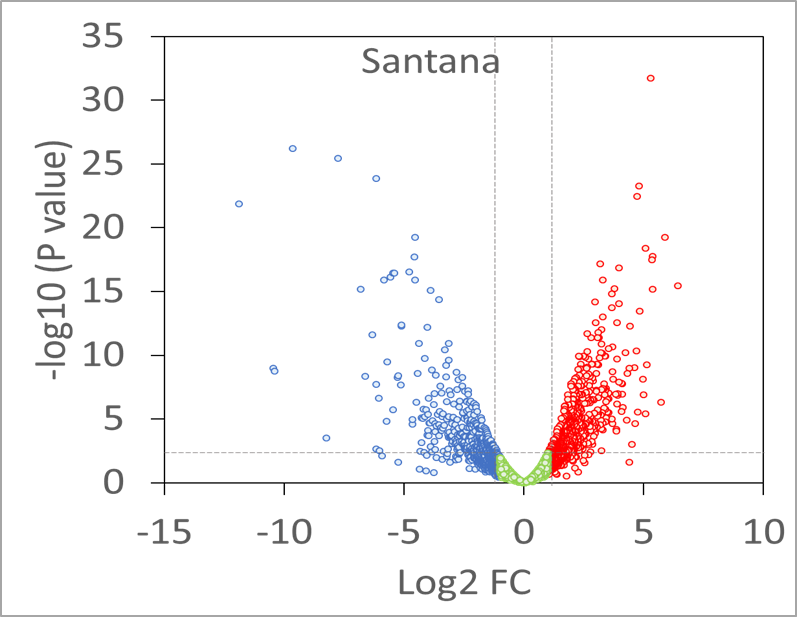

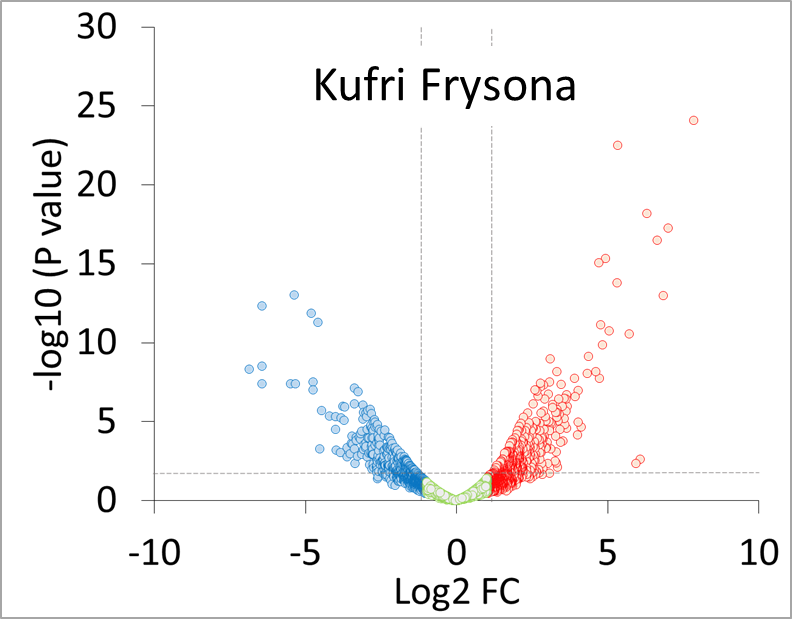

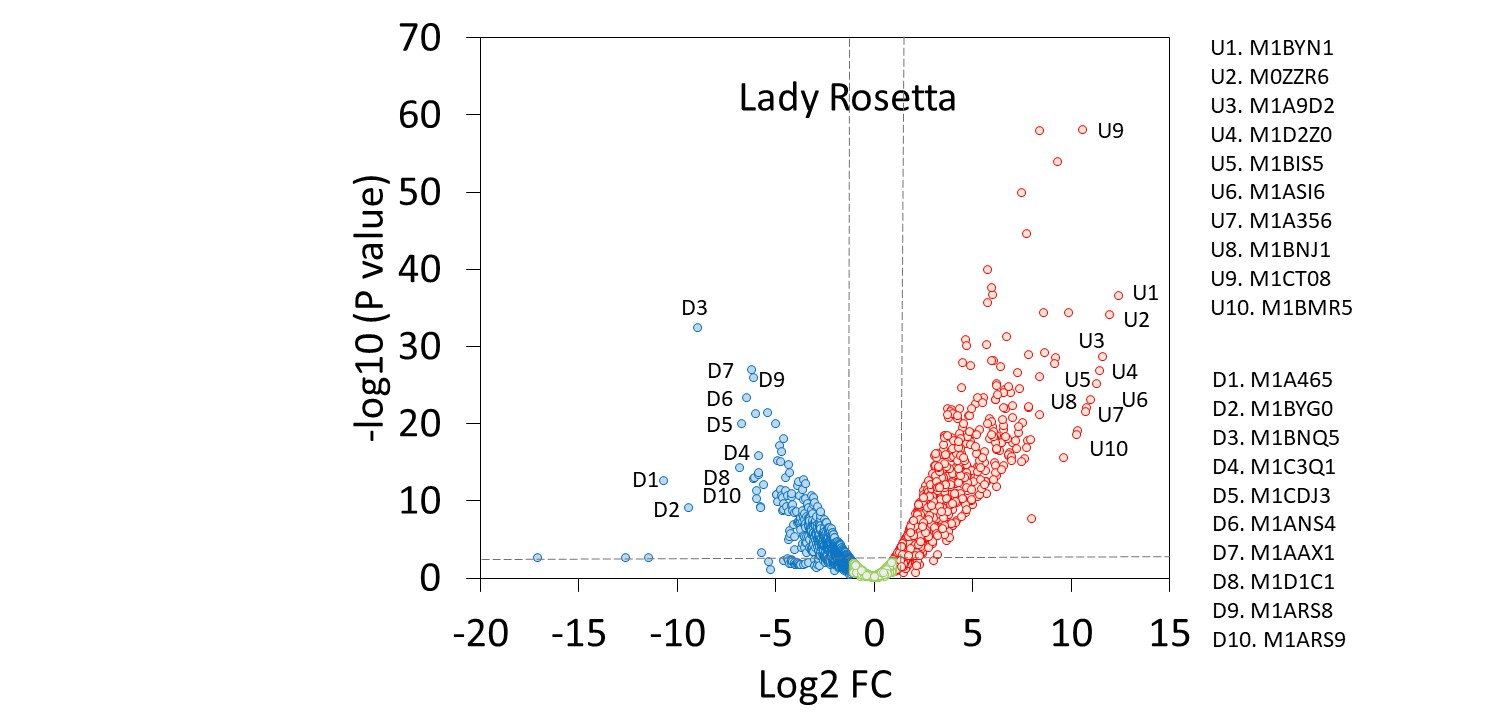


(A) (B) (C)

**Figure S1** Volcano plot showing number of differentially expressed genes in three radiation treated potato varieties compared to their respective non-treated counterparts. The log 2 fold changes (FC) and their corresponding-log 10 (p value) of the annotated genes were taken into consideration for construction of the plot; and the genes with p < 0.05 are presented below the horizontal dotted line. Vertical lines are drawn at positive and negative fold change of ≥ 2. Therefore, red dots on the right hand side of the vertical line and the blue dots on the left hand side mark only above the P <0.05 designate the up-regulated and down-regulated genes, respectively.


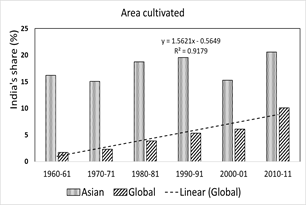

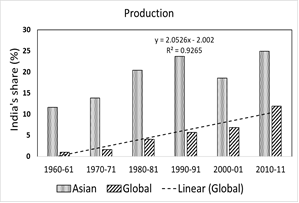

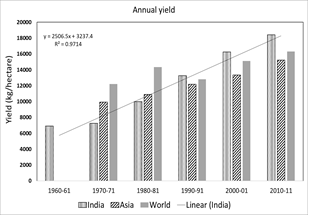

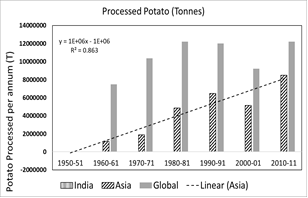

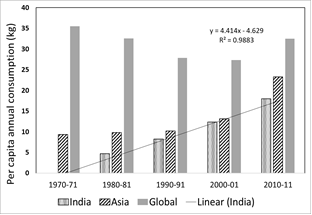


**Figure S2** Potato cultivation area (A), total annual production (B), yield (C), total processed potato (D) and per capita annual consumption ( E): Global, Asian and Indian scenario (Based on data available at www.potatopro.com accessed on 20-4-2023).


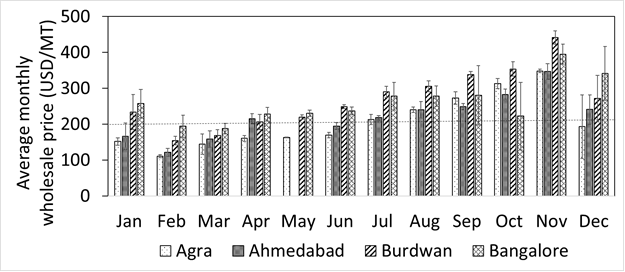


**Figure S3** Annual price variation in Indian wholesale potat markets (Mandis). North zone: UP: Agra Market, West zone: Gujrat: Ahmedabad Chimanbhai Patal Market Vasana, East zone: West Bengal: Burdwan Market; Karnataka (Bangalore) in South zone. Calculated based on data available at agmarknet.gov.in.
